# Oxidative and Inflammatory Mechanisms Induced by Intermittent Hypoxia Leading to Vascular Alterations in Rodents: A Systematic Review and Meta‐Analysis

**DOI:** 10.1155/omcl/9967028

**Published:** 2026-01-14

**Authors:** Marc Adrien Reveyaz, Célian Peyronnel, Quentin Boëte, Joey Fournier, Claire Arnaud, Elise Belaïdi, Olfa Harki, Jean-Louis Pépin, Charles Khouri, Gilles Faury, Anne Briançon-Marjollet

**Affiliations:** ^1^ HP2 Laboratory, CHU Grenoble Alpes, Université Grenoble Alpes, INSERM, Grenoble, 38000, France, univ-grenoble-alpes.fr; ^2^ Pharmacovigilance Unit and Clinical Pharmacology Department, Grenoble Alpes University Hospital, Grenoble, France, chu-grenoble.fr; ^3^ LBTI UMR 5305, Laboratory of Tissue Biology and Therapeutic Engineering, University Lyon 1, CNRS, Lyon, 69367, France, univ-lyon1.fr

**Keywords:** apoptosis, inflammation, intermittent hypoxia, meta-analysis, oxidative stress, systematic review, vascular alterations

## Abstract

**Objectives:**

Obstructive sleep apnea (OSA) and the related intermittent hypoxia (IH) are recognized as major cardiovascular risk factors. In a previous meta‐analysis, we confirmed the impact of IH on structural and functional remodeling of vessels in rodent models of IH. Here, we conducted a systematic review and meta‐analysis to investigate the molecular mechanisms related to vascular remodeling induced by IH and to analyze the impacts of patterns of exposure on the effect of IH.

**Methods:**

We searched PubMed, Web of Science, and EMBASE and included 52 articles, among them 44 concerning wild type (WT) rodents and eight concerning apolipoprotein E knockout (ApoE^−/−^) mice. We used standardized mean difference (SMD) to compare results between studies. A hypoxic score was designed and calculated, and metaregressions were performed to explore the impact of IH exposure parameters on the selected outcomes.

**Results:**

IH induced an increase in oxidative stress, inflammation markers, leucocyte infiltration, and apoptosis, and a decrease in endothelial nitric oxide synthase (eNOS) expression and activity in arteries of WT mice. In metaregressions, inflammation and oxidative stress markers were associated with total duration of IH exposure, and eNOS was associated with hypoxic score. In ApoE^−/−^ mice, inflammation markers were significantly increased in atherosclerotic plaques, but leukocyte infiltration and oxidative stress were not modified by IH. Rodent characteristics had only few impacts on the outcomes.

**Conclusions:**

Our meta‐analysis confirms that IH, independently of measured confounders, has a major impact on oxido‐inflammatory mechanisms in vessels, and that the duration of IH can modulate these effects. Our findings strengthen our understanding of molecular mechanisms associated with vascular alterations in IH/OSA.

## 1. Introduction

Obstructive sleep apnea (OSA) is characterized by repeated breathing interruptions during sleep, caused by upper airway closures often due to surrounding tissue collapse. These episodes, ranging from a few to over 30 per hour [1], pose a significant public health challenge affecting up to 1 billion people globally with daytime sleepiness, accidents, and negative impacts on other serious diseases. OSA is now recognized as a significant risk factor for cardiovascular and metabolic diseases, including hypertension, atherosclerosis, myocardial infarction, stroke, and type 2 diabetes [2], which all appear related to the intermittent hypoxia (IH) it generates [1, 3]. While the apnea‐hypopnea index (AHI), that is, the number of apnea and hypopnea events per hour of sleep, remains the standard for assessing OSA severity, recent studies increasingly suggest that new indexes such as the hypoxic burden (HB) or the sleep breathing impairment index (SBII), both related to nocturnal hypoxic load, may better reflect cardiovascular risk [4–8].

Because many of the extracellular, cellular, and molecular mechanisms leading to OSA impacts on cardiovascular health or outcomes are often difficult to investigate in patients, studies using rodent models have been used over the last few decades to explore IH–related cardiac and vascular impacts. Our two recent meta‐analyses about the structural and functional consequences of IH on blood vessels and heart in rodent models revealed that (i) IH elevates blood pressure, thickens artery walls, impairs vascular reactivity/motricity (at least, in part, through endothelial dysfunction), and promotes atherosclerosis [9], and (ii) IH induces cardiac dysfunction and fibrosis and increases sensitivity to myocardial infarction, notably with severe hypoxia being harmful, while moderate hypoxia (fraction of inspired oxygen [FiO_2_] > 7%, <5 h/day) offers heart protection [10].

The mechanisms by which the OSA/IH mediates vascular structure‐function changes have also been explored. Oxidative stress, inflammation, and nitric oxide (NO) dysregulation in blood vessels have been found to positively correlate with OSA/IH [1, 2, 11, 12], suggesting that these effects might be key factors in understanding OSA–related impacts on cardiovascular disease. The dysregulation of the pro‐oxidant/antioxidant balance by IH results in increases in both reactive oxygen species (ROS) and reactive nitrogen species (RNS), leading to increases of plasma biomarkers of oxidative stress including malonedialdehyde (MDA, a lipid peroxidation end‐product), isoprostanes, advanced oxidation protein products (AOPPs), and DNA oxidation products (8‐hydroxy‐2‐deoxyguanosine, 8‐OHdG) [12]. This oxidative stress may trigger vascular inflammation, characterized by activated leukocytes and endothelial cells and increased proinflammatory transcription factors and cytokines such as nuclear factor kappa B (NF‐*κ*B), tumor necrosis factor (TNF‐*α*), and interleukin (IL) ‐6 [1, 2, 12]. Oxidative stress and inflammation may then, in turn, activate downstream signaling pathways leading to the increased apoptosis of endothelial cells observed in OSA patients [13] and to altered endothelial NO synthase (eNOS) expression and activity, leading to reduced NO bioavailability [12, 14] which, together with eNOS uncoupling, further contributes to increased oxidative stress [15, 16].

There are, however, some notable differences observed between rodent studies related to the impacts of experimental IH on oxidative stress, inflammation, and/or eNOS expression. Such differences could be due, in part, to variability in the impact of IH resulting from endogenous/biological variability between subjects or to methodological reasons. In particular, IH exposure duration appears to be a major determinant of the nature and magnitude of the impact of IH. For example, Zhou et al. [17] demonstrated that eNOS expression was increased after 3 days of IH but was not different than the normoxia controls (*N*) after 1 or 3 weeks of IH exposure and then ultimately decreased after 8 weeks of IH. By contrast, in the same study, biomarkers of oxidative stress (TNF*α* and 3‐nitrotyrosine) were only increased after 8 weeks of IH and were not significantly different than controls at the earlier time points [17]. Philippi et al. [18] performed a time‐course study over 3–56 days of IH and observed that the impact of IH on vessel diameter, collagen deposition, and eNOS expression was variable depending on the duration of IH exposure. In addition to variability related to IH duration, differences between studies might also be explained by the different patterns of IH exposure. For example, in two studies which both used mice exposed to 28 days of IH, the opposing effects on eNOS expression observed might be explained by the difference in both the hypoxia severities (10% vs. 5%) and the IH cycle durations (1 vs. 3 min) that were used [19, 20].

For these reasons, we conducted this systematic review and meta‐analysis in order to clarify the respective importance and impact of the cellular mechanisms in the various rodent IH models, including effects on oxidative stress, inflammation, apoptosis, eNOS expression, and function in arteries, and to explore, using subgroup analyses and metaregressions, the main factors involved in the heterogeneity of effects reported, including those related to the rodent models and IH cycle patterns that were used.

## 2. Methods

The protocol for the meta‐analysis was recorded on PROSPERO under the number CRD42020169940 (https://www.crd.york.ac.uk/prospero/display_record.php?ID=CRD42020169940). Owing to the large amount of available data, our previous publication specifically reported on the impacts of IH on structural and functional vascular outcomes [9], and the remaining data related to the potential cellular mechanisms underlying these vascular outcomes is reported in the present meta‐analysis.

### 2.1. Search Methods and Study Selection

We searched PubMed, Web of Science, and EMBASE for articles published through March 31, 2025, using the terms “Intermittent hypoxia” AND “Rodent” OR “mice” OR “rat” (see PROSPERO for the exact query). We also searched for related keywords and MeSH terms. After the initial search, we screened titles and abstracts to identify relevant articles. Eligibility was based on studies written in English that examined systemic artery outcomes of IH in rodents related to oxidative stress, inflammation, or apoptosis. Full manuscripts were then reviewed for inclusion and exclusion criteria, with two authors independently screening references. Discrepancies were resolved through intrateam discussion.

We included only controlled studies on rodents exposed to chronic IH compared to normoxic controls (21% FiO_2_). IH was defined as repeated hypoxia‐reoxygenation cycles within a day, and chronic IH as the repetition of these cycles for at least 1 day. Wild type (WT) rodents—mice or rats, male or female, age ≥ 4 weeks, and lean or obese—were included. We excluded studies with continuous hypoxia (no normoxia), hypoxia combined with hypo/hypercapnia, those using hypo/hyperbaric conditions, prenatal or perinatal exposure, and transgenic animals, except for apolipoprotein E knockout (ApoE^−/−^) mice used for atherogenesis studies. Studies involving pulmonary or cerebral vessels were also excluded.

Mandatory outcomes were assessment of oxidative stress, inflammation, leukocyte infiltration, apoptosis, or eNOS (expression, phosphorylation, or activity) in arterial tissues (blood measurements were excluded). Oxidative stress markers included dihydroethidium (DHE), 4‐hydroxy‐2‐nonenal (4‐HNE), MDA, superoxide anions, nitrotyrosine, AOPP, or pro/antioxidant enzymes (NADPH oxidase [NOX], xanthine oxidase, superoxide dismutase [SOD], catalase, or glutathione peroxidase). Inflammation markers included mRNA or protein expression of IL‐6, TNF‐*α*, IL‐1*β*, interferon gamma (IFN*γ*), monocyte chemoattractant protein‐1 (MCP‐1), intercellular adhesion molecule‐1 (ICAM‐1), NF‐*κ*B, inducible NO synthase (iNOS), or cyclooxygenase 2 (COX‐2). Apoptosis markers include TUNEL staining, or expression of caspase‐3, B‐cell leukemia/lymphoma 2 protein (BCL‐2), or Bcl‐2‐associated X protein (BAX). Leukocyte infiltration and atherosclerotic plaques were evaluated in WT and ApoE^−/−^ mice.

### 2.2. Assessment of a Hypoxic Score

To mirror the sleep‐apnea patient‐specific HB in animal studies, we developed a self‐referential score, reflecting the characteristics of the IH exposure in the set of the studies selected. While we recognized that the HB used in clinical settings for patients with OSA largely relies on O_2_ desaturation, this was not available in the vast majority of animal studies. Accordingly, we developed a “hypoxic score,” with the aim to provide the best possible quantification of the cumulative intensity of hypoxia exposure based on accessible characteristics common to all the included animal studies. This hypoxic score included the following five parameters: (1) FiO_2_ during hypoxia (X1), (2) hypoxic phase duration (X2), (3) reoxygenation phase duration (X3), (4) daily hypoxic exposure time (X4), and (5) total exposure duration (X5). We assumed that a lower FiO_2_, a greater duration of the hypoxia phase, or an increase in the number of days of exposure all increased the hypoxic load, while a longer reoxygenation duration would likely be a mitigating factor of hypoxic load. We thus categorized each of the five hypoxic exposure parameter values into terciles or halves based on study data, assigning a score from 1 (lowest) to 3 (highest) for each parameter. The hypoxic score was thus calculated as X1 + X2 − X3 + X4 + X5, with X3 (reoxygenation duration) subtracted as it, again, is theoretically a mitigating factor that opposes the hypoxia load. A higher score indicates a more severe hypoxic load (Supporting Information 1 and Supporting Information 2: Figure S8).

### 2.3. Methodological Quality Assessment

Study quality was assessed using the SYRCLE tool [21]. This tool evaluates selection bias (sequence generation, baseline characteristics, and allocation concealment), performance bias (randomized animal housing and investigator blinding), detection bias (random outcome assessment and blinding of assessors), attrition bias (incomplete data), and reporting bias (selective reporting). Bias risk is rated as high, low, or unclear. Four authors independently scored the studies, and disagreements were resolved through consensus.

### 2.4. Statistical Analyses

We conducted separate meta‐analyses for WT rodents and ApoE^−/−^ mice, using standardized mean difference (SMD) and 95% confidence intervals (CIs) for each outcome. An SMD >0.8 was considered large, 0.5–0.8 moderate, and 0.2–0.5 small [22]. If standard deviations (SDs) were missing, we estimated them from CIs or standard errors [23] or imputed them using SD from the other arm when reported. If no SD was reported in any arm, the study was excluded from the analysis. Results were visualized with Orchard plots, an innovative data visualization tool well adapted for displaying the results of a large number of outcomes (Supporting Information 2: Figure S1). For consistency, we inverted SMD signs for antioxidants (SOD and catalase) and antiapoptotic (BCL‐2) and excluded studies with extreme SMD values (>10 or <−10) to limit outlier influence. Random‐effects meta‐analyses were performed using the restricted maximum‐likelihood estimator, with hierarchical models for multiarm studies. For outcomes with >10 studies, we explored heterogeneity via subgroup analyses and metaregressions based on population characteristics (age, sex, and weight) and IH protocols (FiO_2_, phase durations, and exposure time). Metaregressions for WT rodents also accounted for species (rat/mice). Funnel plot asymmetry for primary outcomes was assessed with Egger’s test (*p* < 0.1 for publication bias). We performed a sensitivity analysis by excluding studies with missing and imputed SDs.

Analyses were performed using R statistical software (v4.1.1).

## 3. Results

**Figure 1:**
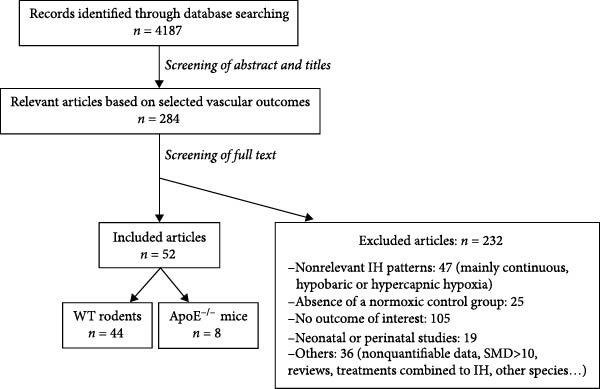
Flow diagram of the study. IH, intermittent hypoxia; WT, wild type. ApoE^−/−^, apolipoprotein E knockout.

Our systematic literature review yielded 4187 references through March 31, 2025, among which we ultimately selected 52 articles for inclusion in the meta‐analysis, including 44 studies in WT rodents and eight studies in ApoE^−/−^ mice (Figure 1). Supporting Information 3: Table S1, Supporting Information 2: Figure S2, and Supporting Information 1 present vascular outcomes available across the studies, the settings of hypoxic exposure, and the experimental designs.

### 3.1. Impact of IH on Inflammation in WT Mice

We examined inflammation markers (gene/protein expression and protein activity) in WT mice, including IL‐6, TNF*α*, IFN*γ*, MCP‐1, IL‐1*β*, TGF*β*, ICAM‐1, NF*κ*B, HIF‐1, iNOS, and COX‐2. IH significantly increased these markers in arterial walls (SMD 2.32 [1.3; 3.33]; Figure 2A and Supporting Information 2: Figure S3A), in parallel with elevated leukocyte infiltration (SMD 2.35 [0.44; 4.26]; Figure 2B and Supporting Information 2: Figure S3B). Following metaregression analyses, leukocyte infiltration correlated with IH exposure duration (slope estimate = 0.03, *p* = 0.022) and with FiO_2_ during hypoxia (slope estimate = 3.04, *p* = 0.048; Table 1 and Figure 2C,D), suggesting that arterial leukocyte infiltration is related to both IH severity and duration. Inflammation markers tended to be associated with duration of the hypoxic phase (slope estimate = −0.03, *p* = 0.064) and with total duration of exposure (slope = 0.01, *p* = 0.081; Table 1).

Figure 2Intermittent hypoxia increases inflammation in systemic vessels of wild type animals. Orchard plots showing standardized mean differences (SMD) for (A) inflammation markers and (B) leukocyte infiltration in arterial walls. (C, D) Association of leucocyte infiltration with total duration of IH (slope = 0.03, *p* = 0.022) and with FiO_2_ (slope = 3.04, *p* = 0.048), respectively. *k* represents the number of effect sizes per estimate.

**Figure:**
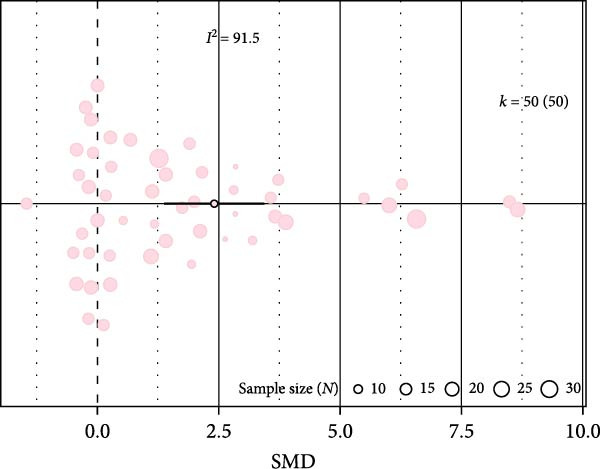
(A)

**Figure:**
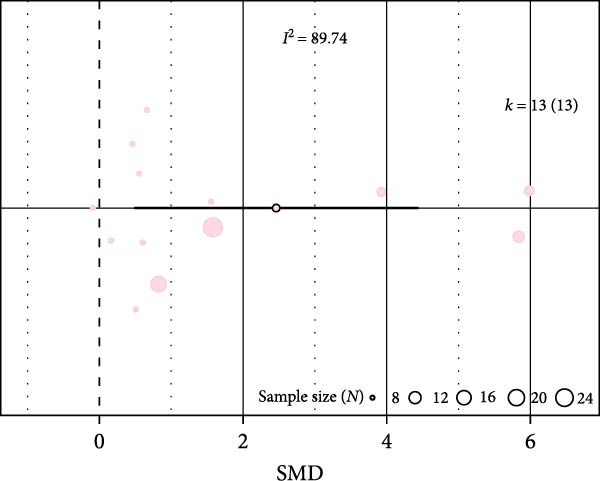
(B)

**Figure:**
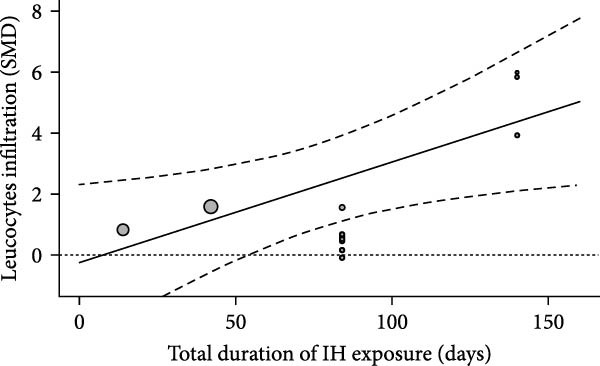
(C)

**Figure:**
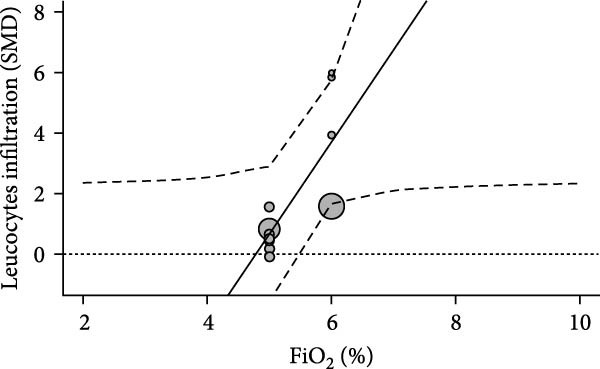
(D)

**Table 1:** Metaregression analyses for the main outcomes: inflammation markers, oxidative stress, eNOS level, leukocyte infiltration in WT animals, and leukocytes infiltration in ApoE^−/−^ mice.

Moderator	Inflammation markers		Oxidative stress		eNOS		Leukocyte infiltration (WT)		Leukocyte infiltration (ApoE^−/−^)	
	Slope	*p*‐value	Slope	*p*‐value	Slope	*p*‐value	Slope	*p*‐value	Slope	*p*‐value
Species	1.01	0.363	0.31	0.746	−1.43	0.262	NA	NA	NA	NA
Age	0.00	0.860	0.00	0.945	0.00	0.964	0.25	0.689	−0.34	0.284
Body weight	−0.02	0.122	−0.01	0.178	0.02	0.006 ^∗^	NA	NA	0.83	0.423
FiO_2_	0.01	0.808	−0.06	0.174	−0.14	0.279	3.04	0.048 ^∗^	−0.30	0.831
Duration of hypoxic phase	−0.03	0.064	0.00	0.785	0.00	0.892	−0.01	0.567	−0.09	0.001 ^∗^
Duration of reoxygenation phase	0.01	0.421	0.00	0.785	0.00	0.929	−0.01	0.567	0.14	0.284
Duration of IH per day	−0.20	0.467	−0.01	0.956	0.21	0.351	0.39	0.497	2.07	0.000 ^∗∗^
Duration of exposure	0.01	0.081	0.02	0.018 ^∗^	−0.05	0.104	0.03	0.022 ^∗^	−0.18	0.158
Hypoxic score	0.30	0.212	0.62	0.004 ^∗^	0.76	0.085	0.45	0.757	0.45	0.834

*Note:* FiO_2_, inspiratory oxygen fraction.

Abbreviation: IH, intermittent hypoxia.

^∗^ *p* < 0.05.

^∗∗^ *p* < 0.001.

### 3.2. Impact of IH on Oxidative Stress, eNOS, and Apoptosis

Oxidative stress was assessed via expression and activity of enzymes (SOD, catalase, NOX, glutathione peroxidase, and xanthine oxidase) and markers including MDA, superoxide anions, oxidized proteins, nitrotyrosine amounts, or DHE staining. IH significantly increased oxidative stress (SMD 1.41 [0.56; 2.25]; Figure 3A and Supporting Information 2: Figure S4A), which correlated with total IH duration (estimate 0.02, *p* = 0.018) and with hypoxic score (estimate = 0.67, *p* = 0.002) in metaregression analyses (Figure 3B,C and Table 1). IH also decreased eNOS expression, phosphorylation, or activity (SMD −1.12 [−2.16; −0.09]; Figure 3D and Supporting Information 3: Figure S4B), which was correlated with body weight in metaregressions (estimate = 0.02, *p* = 0.006) and tended to be associated with hypoxic score (estimate 0.76, *p* = 0.085). Despite the small number of studies, apoptosis levels assessed by TUNEL staining, caspase‐3, BAX, and BCL‐2 expression were significantly elevated after IH (SMD 4.2 [2.5; 5.9]; Figure 3E and Supporting Information 2: Figure S4C).

Figure 3Intermittent hypoxia increases oxidative stress and apoptosis in systemic vessels of wild type animals. Orchard plots showing standardized mean differences (SMD) for (A) oxidative stress, (B. C) correlation between oxidative stress and total duration of IH exposure (days, slope 0.02, *p* = 0.018) and with hypoxic score (estimate 0.67, *p* = 0.002), respectively. (D) eNOS expression and activity and (E) apoptosis in arterial walls. *k* represents the number of effect sizes per estimate.

**Figure:**
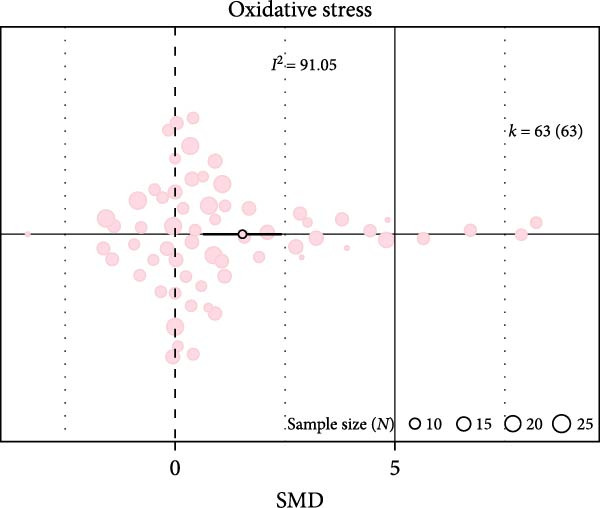
(A)

**Figure:**
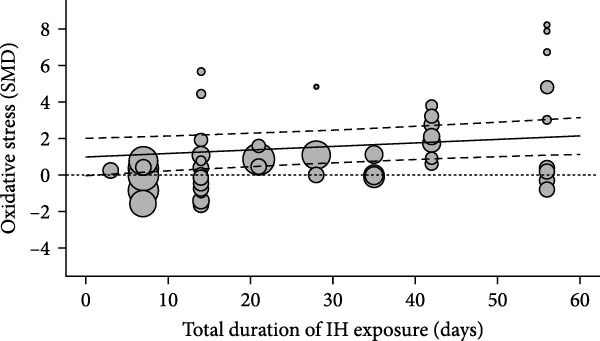
(B)

**Figure:**
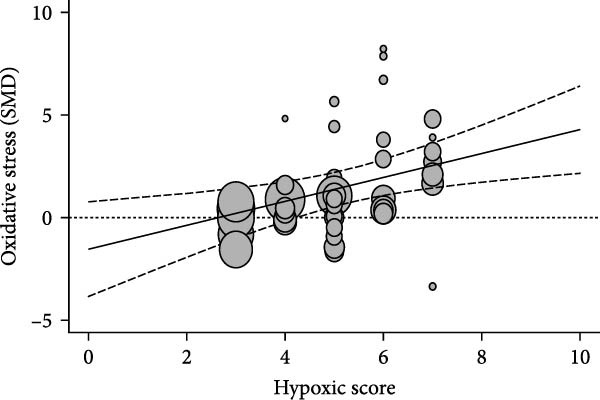
(C)

**Figure:**
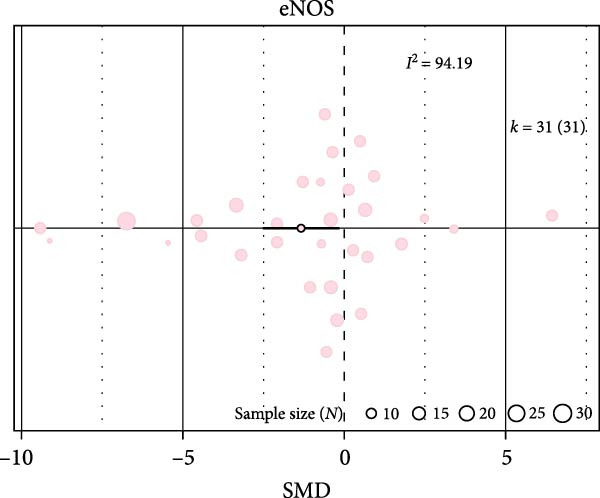
(D)

**Figure:**
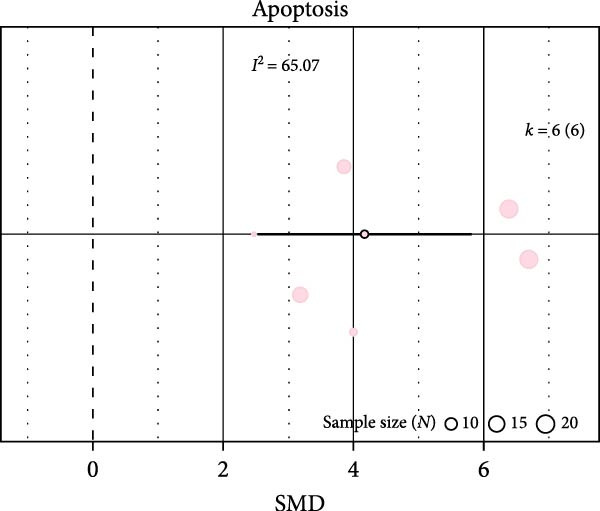
(E)

### 3.3. Impact of IH on ApoE^−/−^ Mice

In ApoE^−/−^ mice, due to the low number of studies, only three outcomes could be analyzed. IH significantly increased inflammation markers (SMD 3.32 [1.25; 5.4]; Figure 4A and Supporting Information 2: Figure S5A) but had no significant effect on leukocyte infiltration (SMD 2.23 [−1.39; 5.85]) or oxidative stress (SMD 2.87 [−2.16; 7.9]; Figure 4B,C, and Supporting Information 2: Figure S5B,C). In metaregressions, leukocyte infiltration correlated with both hypoxic phase duration (estimate 0.09, *p* = 0.001) and daily exposure time (estimate 2.07, *p* < 0.001; Figure 4D and Table 1).

Figure 4Effect of intermittent hypoxia in ApoE^−/−^ mice. Orchard plots showing standardized mean differences (SMD) for (A) inflammation markers, (B) leukocyte infiltration, and (C) oxidative stress. *k* represents the number of effect sizes per estimate. (D) Correlation between leukocyte infiltration and duration of hypoxic phase (slope 0.09, *p* = 0.01).

**Figure:**
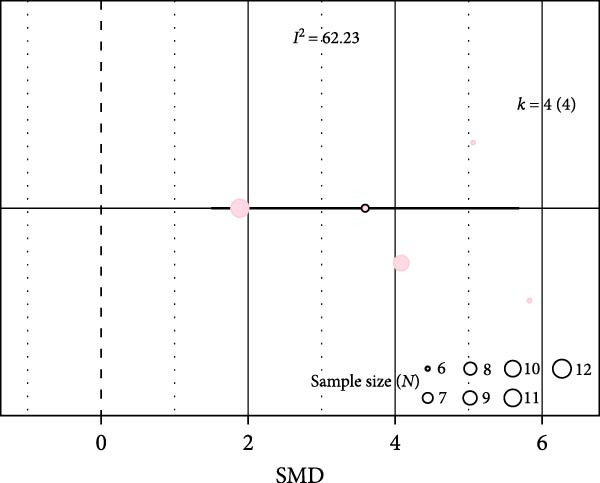
(A)

**Figure:**
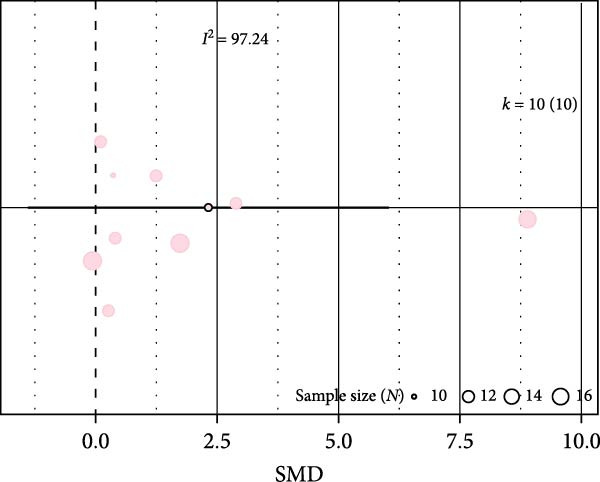
(B)

**Figure:**
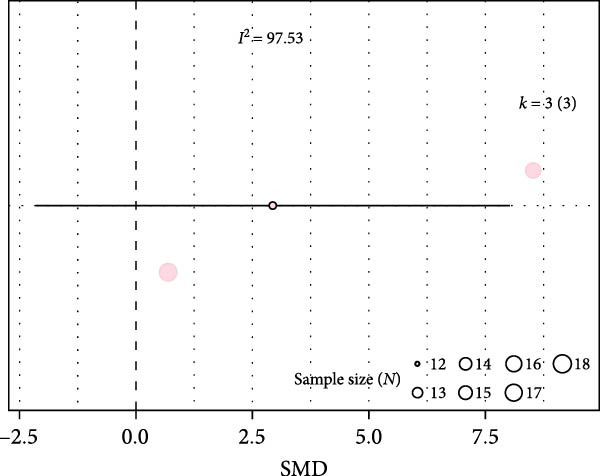
(C)

**Figure:**
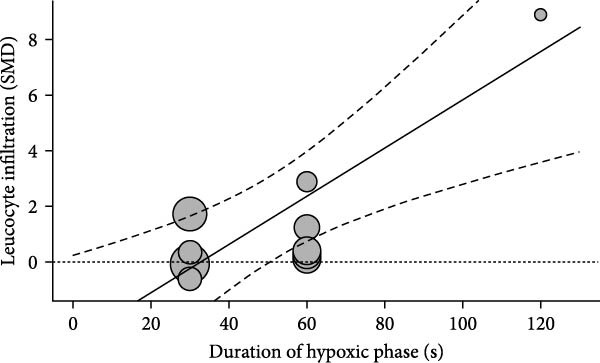
(D)

### 3.4. Risk of Bias and Small Study Effect

Using the SYRCLE tool [21], bias risk was considered as low for 25%–50% of studies for sequence generation, baseline characteristics, incomplete outcome data, and selective outcome reporting. On the other hand, the risk was high for 33% of studies for incomplete outcome data (Supporting Information 2: Figure S6 and Supporting Information 4: Table S2). For all other SYRCLE items, ≥95% of studies scored as unclear as the information was almost never mentioned in publications. Funnel plots revealed asymmetry and significant Egger tests, indicating small study effects (Supporting Information 2: Figure S7).

## 4. Discussion

IH is a major feature of OSA and the major source of the cardiovascular complications associated with this condition. Our previous meta‐analyses reported that IH increased arterial pressure, impaired vasodilation, and induced vascular remodeling, with some result heterogeneity related to the researcher networks [9, 24]. The current meta‐analysis aimed to further explore the impact of IH patterns and severity on major cellular and molecular mechanisms known to drive the vascular structure/function changes: inflammation, oxidative stress, eNOS (activity or expression), and apoptosis. A second objective of the present work was to verify whether the IH–induced modulation of these mechanisms was dependent on hypoxia duration and/or hypoxic score, and therefore, whether the parameters of the IH protocol substantially impacted the results. As a deepening of our previous meta‐analysis of the functional impact of IH on vessels [9], this new meta‐analysis does provide valuable insights into the underlying molecular mechanisms that could represent therapeutic options to treat vascular dysfunction in OSA patients.

### 4.1. Impact of IH on the Vascular Molecular Mechanisms Targeted in the Present Meta‐Analysis

Our results show that IH is associated with a significant increase in inflammation (including leukocyte infiltration) and oxidative stress markers in arterial walls of WT rodent models, consistent with systemic inflammation and oxidative stress seen in OSA patients [1, 2, 25, 26]. Despite a low number of studies reducing the level of evidence, in our meta‐analysis, IH also significantly increased inflammation in atherosclerotic plaques in ApoE^−/−^ mice, supporting previous findings [9] and reinforcing OSA as a major atherosclerosis risk factor [27, 28]. Key inflammation markers such as TNF‐*α* and NF‐*κ*B and oxidative markers such as nitrotyrosine residues and NOX expression/activity were frequently reported to be increased in vessels or blood of OSA patients [29–33], while antioxidant defenses such as SOD were diminished [34, 35]. In the rodent models and the studies included in the present meta‐analysis, the simultaneous rise in oxidative stress and inflammation supports the view of an intertwining of these two mechanisms through mutual stimulation, as widely described in the literature [1, 11, 36]. Moreover, oxidative stress was associated with total duration of exposure to IH and to the hypoxic score, while leukocyte infiltration was associated with FiO_2_ and duration of exposure. These results point out the impact of the hypoxic load and, in particular, the duration of IH exposure on the stimulation of molecular and cellular mechanisms associated with the progression of cardiovascular disease in OSA. This is consistent with the findings from our previous meta‐analysis in rodent models, showing that the increases in mean arterial pressure and intima–media thickness after IH exposure tend to correlate with IH exposure duration [9]. From a technical point of view, our meta‐analysis, thus, suggests that experimental designs with longer IH exposure and more severe IH patterns may be more appropriate to explore OSA–related cardiovascular pathophysiology. In clinical practice, this suggests that the time‐course of the disease, which may be hard to evaluate in many newly diagnosed OSA patients, could be an important factor associated with the severity of vascular disease. Our results are also consistent with and strengthen recent findings in patients that the severity of hypoxia—as evaluated by the HB or by the SBII [4–8]—may be the most relevant determinant to evaluate the severity of the cellular, molecular, and functional consequences of OSA on the cardiovascular system.

Regarding NO signaling, the present meta‐analysis revealed that IH significantly reduces eNOS expression or activity, consistent with the impaired acetylcholine‐induced vasodilation present in IH‐exposed mice, as demonstrated in our previous meta‐analysis [9]. This also mirrors the clinical finding of reduced NO bioavailability in OSA patients [12, 14, 16]. Hypoxic conditions in OSA contribute to the depletion of the eNOS cofactor tetrahydrobiopterin (BH4) and the eNOS substrate L‐arginine, leading to eNOS uncoupling—a state favoring superoxide anion instead of NO production by eNOS [15, 16, 37]. Accordingly, eNOS uncoupling may thereby contribute to oxidative stress and reduced NO bioavailability, and the additional activation of iNOS in pro‐oxidant conditions, resulting in the generation of peroxynitrites, and thereby producing nitrosative stress, may further exacerbate endothelial dysfunction [38]. In OSA patients, reduced NO bioavailability parallels endothelial dysfunction evidenced by the observed decreased flow‐mediated dilation and altered reactive hyperemia index [39, 40]. Together, these results highlight the importance of studying the association between endothelial dysfunction and HB in humans with OSA. Finally, while our study demonstrates a clear impact of IH per se on mechanisms regulating endothelial function, some new results suggest that sleep fragmentation could be an added stimulus leading to further alteration of endothelial dysfunction in both mice and OSA patients [41, 42].

Although we could not perform metaregressions due to the small number of studies, we did find that IH increased apoptosis in arterial walls. Similarly, in OSA patients, very few studies have addressed vascular apoptosis. However, consistent with our meta‐analysis, Jelic et al. [13] reported elevated apoptosis of venous endothelial cells in OSA patients compared to controls and a reduction of apoptotic rate after CPAP treatment.

### 4.2. Impact of the IH–Induced Molecular Mechanisms on the Vascular Phenotype: Causality Links

Depending on the HB, IH substantially alters the arterial structure (wall thickness, elastic fiber/lamella integrity, collagen organization, atheroma plaques, etc.) and function (endothelium permeability, endothelial vasoactive agent production, smooth muscle cell reactivity, arterial stiffening, etc.) through the activation or deactivation of a series of molecular pathways [9, 18, 43, 44]. As shown in the present meta‐analysis, IH induces an increase in oxidative stress, inflammation markers/leukocyte infiltration, and apoptosis, and a decrease in eNOS expression or activity in mouse arteries. These results are particularly compelling since they align with a number of previous reports demonstrating that these same molecular mechanisms are causal in some or all of the above‐listed IH–induced phenotypic changes observed in arteries.

Regarding leukocyte infiltration, IH has been demonstrated to stimulate the secretion of the leukoattractant cytokine regulated upon activation, normal T‐cell expressed and secreted (RANTES)/CC chemokine ligand 5 (CCL5), which is known to be critically involved in early atherogenesis. In mice with IH–induced hypertrophy of smooth‐muscle cells, RANTES/CCL5 neutralization prevented both the aorta intima–media thickening and inflammatory alterations otherwise observed [44].

Similarly, regarding the impact of the elevated oxidative stress observed in both OSA patients and rodent IH models, antioxidative treatments have been shown to limit or abolish the endothelial and vascular smooth muscle cell dysfunction, as well as vascular remodeling and increase in blood pressure observed [11, 45–49].

Regarding the role of apoptosis, it has been shown that the aortic wall remodeling and endothelial cell apoptosis, induced by IH in rats, were reversed by antiapoptotic treatment [50]. And finally, with regard to the role of eNOS, it has been clearly demonstrated that, by reducing NO bioavailability and promoting a pro‐oxidant state, IH–associated eNOS uncoupling and reduced activity contribute to endothelial dysfunction and hypertension in rodent models of IH [9, 20, 51].

Together, these studies demonstrate that IH–induced vascular inflammation, oxidative stress, eNOS uncoupling, and apoptosis are indeed involved in the observed IH–induced changes in vascular structure and function.

### 4.3. Contribution of Other IH–Induced Molecular/Cellular Mechanisms

Our meta‐analysis was limited to a reduced number of vascular outcomes, for which sufficient studies could be identified in the literature to perform the meta‐analysis. However, several other molecular or cellular mechanisms may also participate in the IH–induced vascular alterations. Among them, endothelial activation and increased permeability may be early steps of vascular remodeling and atherosclerosis induced by IH involving multiple signaling pathways including catecholamines [52], VE–cadherin cleavage related to the VEGF and Src kinases signaling pathways [43, 53], ERK and JNK MAP kinases [54], NF‐*κ*B [55], and the nonmuscle myosin light chain kinase (nmMLCK) [56]. IH impacts have also been demonstrated on a number of other major molecular pathways controlling vascular cell survival and function and extracellular matrix (ECM) maintenance/degradation. First, in rats, IH was shown to modulate intracellular calcium homeostasis in vascular smooth muscle cells, leading to increased Ca^2+^ wave frequency in arteries stimulated by endothelin‐1, compared to the arterial response in normoxic animals [57]. Second, IH has been shown to generate endoplasmic reticulum stress, leading to increased endothelial cell apoptosis, both in a rat model and in vitro [58]. Third, in mice, IH was demonstrated to increase vascular cell production of matrix metalloproteinases (MMPs) and metalloproteinases, including ADAM‐17 and the elastases MMP2 and MMP9, resulting in alteration of elastin in the aortic wall and the development of aortic aneurysms [59]. Finally, the sympathetic nervous system appears to have a significant influence on vascular remodeling and blood pressure in both OSA and IH models [60–62].

These alterations in signaling pathways are primarily driven by a disruption of the transcription factor network by IH, notably through the upregulation and stabilization of HIF‐1*α* [19, 63], while knockdown of HIF‐1*α* in vivo abolishes the vascular remodeling induced by IH [64]. Notably, HIF‐1*α* also crosstalks with several key signaling molecules involved in inflammatory and oxidative stress responses, including NF‐*κ*B, as well as components of the MAPK and nuclear factor (erythroid‐derived 2)‐like 2 (NRF2)/heme oxygenase‐1 (HO‐1) pathways [64–67]. IH–induced alterations in microRNA expression, including miR‐21, miR‐23a‐3p, or miR‐181a‐1, also interact with HIF‐1*α*, NF‐*κ*B, and the MAPK signaling pathway to regulate endothelial activation, inflammation, autophagy, and apoptosis [67–69]. IH also triggers epigenetic modifications, including changes in DNA methylation and shifts in the acetylation/deacetylation balance, further promoting HIF‐1*α* stabilization and potentially leading to differential gene regulation/dysregulation that contributes to vascular dysfunction [70].

### 4.4. Calculation of a Hypoxic Score

In this study, we proposed a composite hypoxic score, designed to assess the severity of IH exposure in experimental models. This score integrates five key components commonly reported in the literature: (1) the duration of the hypoxic phase within each hypoxia‐normoxia cycle, (2) the total number of days of IH exposure, (3) the FiO_2_ during hypoxia, (4) the cumulative duration of hypoxia per day, and (5) the duration of reoxygenation phases. The calculation of this hypoxic score is based on pooled data and stratified using terciles or halves, making it an autoreferential index which reflects the set of included studies. One of its key advantages is the incorporation of variables that are often inaccessible in clinical settings—such as the total duration of IH exposure over days or weeks—which may be critical for understanding the full pathophysiological impacts of IH. The metaregression analysis using the hypoxic score yielded results distinct from those obtained with each individual component, suggesting that this composite score is nonredundant and provides additional explanatory power. In addition, existing literature has shown that the metabolic and cardiovascular deleterious effects of IH are exacerbated by lower FiO_2_, higher frequency of hypoxic phases per day, and longer total exposure duration [9, 10, 71, 72]. In line with our findings, our previous meta‐analysis examining the cerebral consequences of IH demonstrated significant associations between neuronal (e.g., BDNF) and oxidative stress biomarkers (e.g., MDA and NOX) with specific parameters of the IH exposure protocol—namely, the total duration of exposure, the duration of the hypoxic phase, and the FiO_2_ [73]. These results underscore a critical role of individual IH exposure parameters in modulating molecular responses to IH and the importance of trying to quantify their composite impact.

However, the hypoxic score has several limitations. First, it is autoreferential, with thresholds derived from the distribution of values in the meta‐analysis dataset. Thus, the score must be recalibrated for use in single studies or alternative data pools. Second, due to the lack of real‐time oxygenation data in rodent models, the score cannot incorporate an actual area under the curve for arterial oxygen partial pressure (PaO_2_) or peripheral oxygen saturation (SpO_2_), a key measure of HB used in clinical practice. Future refinement could address these limitations through the integration of telemetry‐based physiological data, enabling 24‐h recordings of PaO_2_/SpO_2_, blood pressure, or other dynamic parameters. Third, the relative contribution of each component remains to be clarified; for example, it is still uncertain whether FiO_2_ has a more deleterious effect than cumulative exposure duration or whether prolonged reoxygenation is truly protective or potentially detrimental. This highlights the need to design dedicated studies aiming to optimize the scoring system by assessing the individual impact of each of the IH exposure parameters on well‐defined clinical outcomes such as blood pressure or intima–media thickness.

At this stage, the proposed score should be regarded as a preliminary conceptual framework, presenting significant limitations that must be overcome. Nevertheless, following appropriate refinement and validation, this score could provide the foundation for a standardized tool, thereby enabling more consistent assessments of hypoxia severity across studies in animals and facilitating interstudy comparisons.

### 4.5. Contribution of Rodent Model Characteristics to the Impact of IH

Our metaregressions showed that eNOS expression was associated with body weight (after adjusting for rat/mouse species), suggesting that eNOS is decreased in animals with low body weight.

Other characteristics of animals, such as age, did not have any significant impact on the results, although most of the studies used young C57Bl/6 mice (up to 12 weeks). The small number of aged mice limits the assessment of age‐related effects on IH‐induced vascular parameters. Similarly, only a small number of studies included female rodents, which prevented sex‐related metaregression analyses. Only two of the studies used both genders, and ApoE^−/−^ males and female mice were pooled without information available on specific data for each sex, thus, preventing a comparison between sexes [74, 75]. Two other studies in our database, which included only females, had conflicting results with elevated or decreased oxidative stress in rat aortas exposed to IH [76, 77]. Notably, our previous meta‐analysis showed IH–induced vasodilation impairment in males, but not females [9], and others have previously reported protection from IH–triggered vascular intima–media thickening in ApoE^−/−^ female mice [78]. In rodents, sex‐dependent effects of IH have also been demonstrated on circulating inflammatory cytokines and oxidative stress [79] as well as on neural function [80, 81] and on metabolism [82]. This is consistent with well‐documented gender differences in OSA, female patients presenting with different clinical features (more atypical symptoms such as insomnia, morning headaches, and nocturia), increased susceptibility to adverse cardiovascular outcomes including hypertension, especially in severe OSA patients, and elevated systemic inflammation [83, 84]. Moreover, apart from cardiovascular outcomes, other conditions such as diabetes mellitus, thyroid disease, and asthma seem to be more frequent in female patients [83, 84]. Nevertheless, OSA remains an underdiagnosed and undertreated cardiovascular risk factor in female patients [83, 85, 86]. The lack of preclinical studies on female rodents reflects the underrepresentation of women in clinical studies, and future studies are thus needed comparing IH impacts in males and females to advance our understanding of the importance of gender on IH–induced vascular cell changes and mechanisms. New ethical recommendations favoring animal experimentations in both sexes, as well as increasing awareness for the necessity of improving female OSA patients’ diagnosis and care, should greatly improve our knowledge concerning sex effects in IH in the coming years.

### 4.6. Risk of Bias and Limitations of the Study

As commonly found in rodent meta‐analyses [9, 10, 87], SYRCLE quality analysis revealed a lack of quality and/or insufficient reporting of information in many studies, underlining the need for standardized reporting and more robust approaches including randomization and blinding. Moreover, animal studies, which generally include only a small number of subjects, may lead to important variability and/or small study effect, as evidenced by the asymmetric funnel plots and significant Egger regression for the outcomes studied here, suggesting possible publication bias. In some studies, SDs were not reported, thus, reducing the quality of the meta‐analysis. A sensitivity analysis was performed which demonstrated that exclusion of these studies did not significantly modify the results (Supporting Information 2: Figure S9), suggesting that imputation of the missing SDs did not have an important impact on our findings. Finally, other factors, such as nonreported laboratory conditions and/or author group biases, might also potentially explain some of the heterogeneity observed [24].

Another important point is that, due to the relatively low number of studies for each parameter, we used composite outcomes merging several different molecules, RNA or protein expression, or enzyme activity to compute the impacts on inflammation, oxidative stress, and eNOS, and these pooled composite outcomes may account for some of the variability observed. More generally, our meta‐analysis included only a limited number of studies, in particular for ApoE^−/−^ mice as well as for leukocyte infiltration and apoptosis in WT mice. The results obtained in ApoE^−/−^ mice should, thus, be interpreted with caution and also because only three parameters could be meta‐analyzed in this population. More studies are thus needed to gain sufficient statistical power for individual meta‐analysis for these parameters. Together with possible biases, interspecies variability, and the rather severe hypoxic protocols used in many studies (corresponding to very severe desaturations when translated to human OSA patients), the significance of some results and the potential translation to the human physiopathological mechanisms related to OSA are limited.

Finally, one of our main objectives was to assess the impact of patterns of IH cycles on the vascular response to IH. However, the variability of the IH protocols between studies was limited with a large number of studies employing alternating 5% FiO_2_/30 s–21% FiO_2_/30 s for 8 h per day, for 2–8 weeks. Further studies with more variability in IH protocols are, thus, needed to more firmly establish the impact of different IH patterns in rodents on vessel parameters and outcomes.

## 5. Conclusions

Consistent with our previous meta‐analysis documenting the impact of IH on vascular functional and structural remodeling, our new work provides robust evidence for a deleterious impact of IH on arterial inflammation, oxidative stress, and eNOS expression. Although this analysis had some limitations, including a low number of studies for some parameters and some potential biases which may moderate the interpretation of our findings, our results highlight the importance IH exposure duration and overall hypoxic score and strongly suggest that IH per se is likely a major contributor to the vascular alterations observed in OSA patients. This meta‐analysis further suggests that future improvement of our present understanding of the consequences of IH on the cardiovascular system, and the mechanisms involved, will require additional studies allowing robust comparisons of different hypoxic loads (mainly through variation of IH severities and/or durations) on selected parameters and outcomes. This will require the use of rigorous methodology, following SYRCLE’s recommendations to limit study biases, and both male and female animals to investigate any sex effect.

## Conflicts of Interest

The authors declare no conflicts of interest.

## Author Contributions

Marc Adrien Reveyaz and Célian Peyronnel should be listed as co‐first authors.

## Funding

This work was funded by the University Grenoble Alpes, the INSERM, the Fondation Agir pour les Maladies Chroniques, the Fondation du Souffle, and the Fondation de l’Avenir pour la Recherche Appliquée.

## Supporting Information

Additional supporting information can be found online in the Supporting Information section.

## Supporting information


**Supporting Information 1** This manuscript includes supplementary methods, supplementary results, two supplementary tables, and 9 supplementary figures in attached files.

**Supporting Information 2** Figure S1: Orchard plot example showing the meaning of the different parts of the plot. Figure S2: Description of the number of studies included for each of the four IH parameters: (A) FiO_2_ during hypoxic phase (in %), (B) duration of hypoxic phase (in seconds), (C) duration of IH exposure per day (in hours), (D) duration of reoxygenation (in seconds), and (E) total duration of IH exposure (in days). Figure S3: Forest plots for (A) inflammation markers and (B) leukocyte infiltration in wild type mice. Figure S4: Forest plots for (A) oxidative stress, (B) eNOS activity, and (C) apoptosis in vascular wall in wild type mice. Figure S5: Forest plots for (A) inflammation markers, (B) leukocyte infiltration, and (C) oxidative stress in ApoE^−/−^ mice. Figure S6: Risk of study bias analyzed with the SYRCLE tool. For each item, the percentage of studies scored low/unclear/high risk of bias is shown. Figure S7: Funnel plots showing publication bias for the main outcomes: inflammation markers (A), eNOS (B), oxidative stress (C) in WT mice, and leukocyte infiltration in ApoE^−/−^ mice (D). The reported *p*‐values correspond to the Egger regression test. Figure S8: Tercile distribution of studies and calculation of hypoxic score. The studies were distributed depending on the settings for each of the five hypoxic cycles parameters: (A) FiO2 during hypoxic phase (in %), (B) duration of each hypoxic phase (in seconds), (C) duration of IH exposure per day (in hours), (D) total duration of IH exposure (in days), and (E) duration of reoxygenation (seconds). The values were divided into two or three parts based on the calculation of halves or terciles and a score between 1 and 3 was assigned according to the distribution of studies into these two or three parts for the five parameters studied, in order to calculate a hypoxic score (F). Figure S9: Forest plot showing the SMDs for the main outcomes after sensitivity analysis (exclusion of the studies with imputed SD).

**Supporting Information 3** Table S1: Full description of included studies, parameters evaluated, and details of hypoxic score calculation.

**Supporting Information 4** Table S2: Syrcle analysis for the risk of bias of included studies.

## Data Availability

The data that support the findings of this study are available from the corresponding author upon reasonable request.
