# Correction to “Oxidative and Inflammatory Mechanisms Induced by Intermittent Hypoxia Leading to Vascular Alterations in Rodents: A Systematic Review and Meta‐Analysis”

**DOI:** 10.1155/omcl/9854762

**Published:** 2026-04-08

M. A. Reveyaz, C. Peyronnel, Q. Boëte, et al., “Oxidative and Inflammatory Mechanisms Induced by Intermittent Hypoxia Leading to Vascular Alterations in Rodents: A Systematic Review and Meta‐Analysis,” *Oxidative Medicine and Cellular Longevity* (2026): 2026 9967028, https://doi.org/10.1155/omcl/9967028.

In the article titled “Oxidative and Inflammatory Mechanisms Induced by Intermittent Hypoxia Leading to Vascular Alterations in Rodents: A Systematic Review and Meta‐Analysis,” information was omitted in the funding section. The corrected section appears below:

**Funding**

This work was funded by University Grenoble Alpes, INSERM, Agence Nationale de la Recherche (ANR, France, grant PASTE: ANR‐25‐CE52‐0614), Fondation Agir pour les Maladies Chroniques, Fondation du Souffle, Fondation de l’Avenir pour la Recherche Appliquée.

We apologize for this error.

