# Supplementary material for: Oxidative and Inflammatory Mechanisms Induced by Intermittent Hypoxia Leading to Vascular Alterations in Rodents: A Systematic Review and Meta‐Analysis
Source: Oxid Med Cell Longev. 2026 Jan 14;2026:9967028. doi: 10.1155/omcl/9967028 (PMC12802560; doi:10.1155/omcl/9967028)
Supplement: Supplementary file 1 — Supporting Information 1 This manuscript includes supplementary methods, supplementary results, two supplementary tables, and 9 supplementary figures in attached files. [file OMCL-2026-9967028-s004.docx]

**Supplementary data**

**Oxidative and inflammatory mechanisms induced by intermittent hypoxia leading to vascular alterations in rodents: a systematic review and meta-analysis.**

**Supplementary methods: assessment of hypoxic score**

For each of the hypoxia parameters assessed in the studies included in the meta-analysis, each value was considered by being assigned to one of the 3 intervals/parts considered (or 2 parts for hypoxia duration per day only), based on the calculation of terciles (Supplementary figure 8). A score was assigned according to the distribution of studies into these 3 parts for each of the five parameters studied (X1, X2, X3, X4, X5), as follows:

- for the duration of the hypoxia phase during each hypoxia-normoxia cycle (X1), the studies received an increasing score corresponding to their position in the 3 parts, as follows: X1 [10s; 30s] = 1; X1 [60s; 90s] = 2; X1 [100; 240 s] = 3;
- for the total number of days (d) of exposure to IH (X2), the studies received an increasing score corresponding to their position in the 3 parts, as follows: X2 [3d; 21d] = 1; X2 [25d; 42d] = 2; X2 [56d; 140d] = 3;
- for FiO_2_ (fraction of inspired oxygen, in %) during the hypoxia phase (X3), this scoring was reversed, considering that the studies corresponding to the first part had the highest O_2_ deprivation: X3 [0; 5] = 3; X3 [5,5; 7] = 2; X3 [7.5; 10] = 1;
- for the duration of hypoxia per day, as the distribution of studies did not allow for separation based on terciles calculation (X4), it was decided to score according to halves, as follows: X4: ≤ 8 hours of hypoxia per day = 1; X4 > 8 hours per day = 2;
- for the duration of reoxygenation (X5), the studies received an increasing score corresponding to their position in the 3 parts, as follows: X5 [15s; 30s] = 1; X5 [40s; 90s] = 2; X5 [110s; 350s] = 3;

In order to obtain a general hypoxic score, we added each previous assigned score for FiO_2_, duration of hypoxic phase, duration of IH per day and total IH exposure duration, and subtracted the duration of reoxygenation score. The hypoxic score, expected to have a value between 1 and 10, was calculated for each study.

*Hypoxic score = (X1 Duration of IH phase + X2 Total IH exposure duration + X3 FiO_2_ during IH phase + X4 Duration of IH per day) - X5 Duration of reoxygenation*

**Supplementary results**

Description of rodent models and IH protocols used in the included studies

Among the 44 studies on wild-type rodents, 23 were performed in mice (22 in C57BL/6 and 1 in 129S1 mice) and 21 in rats (19 in Sprague-Dawley and 2 in Wistar rats). Median body weight (when mentioned) was 24,5 (22.5-36.6) g for mice and 260 (190-435) g for rats. Median age was 8.5 (4-78) weeks in mice and 8 (4-22) weeks in rats, considering that age was not clearly stated in 3 studies in mice and 11 studies in rats. In mice, males were used in 16 studies and females in 2 studies while, in rats, 20 studies used males and only 1 used females. Except for 4 studies that used both standard and high fat diet and 8 studies that did not mention it, the diet was declared as standard in other studies.

Among the 8 analyzed studies performed in ApoE^−/−^ mice, body weight was mentioned in only 2 studies (29 and 30.4 g). Median age was 9.5 (7-21) weeks. Males were used in all studies, including 2 studies in which both males and females were used. The diet was declared as standard in 4 studies and high fat in 4 others.

Regarding IH protocols, median values of FiO_2_ during hypoxic phases were 5.5% (0-10%), the median duration of hypoxic phase was 30 (10-180) s followed by 60 (15-300) s of reoxygenation at 21% of FiO_2_ (Supplementary figure 2)_._ Cycles were repeated, on average, 8h per day for a median duration of 28 (3-140) days. Concerning the hypoxic score, the median value was 5 (3-8) (Supplementary table 1 and supplementary figure 8).

**Supplementary table legends**

**Supplementary table 1:** Full description of included studies, parameters evaluated and details of hypoxic score calculation.

**Supplementary table 2:** Syrcle analysis for the risk of bias of included studies

**Supplementary figures legends**

**Supplementary figure 1:** Orchard plot example showing the meaning of the different parts of the plot

**Supplementary figure 2:** Description of the number of studies included for each of the four IH parameters: (A) FiO_2_ during hypoxic phase (in %), (B) duration of hypoxic phase (in seconds), (C) duration of IH exposure per day (in hours), (D) duration of reoxygenation (in seconds), (E) total duration of IH exposure (in days).

**Supplementary figure 3**: Forest plots for A) inflammation markers and B) leukocyte infiltration in wild type mice

**Supplementary figure 4**: Forest plots for A) oxidative stress, B) eNOS activity and C) apoptosis in vascular wall in wild type mice.

**Supplementary figure 5**: Forest plots for A) inflammation markers, B) leukocyte infiltration, and C) oxidative stress in ApoE^-/-^ mice.

**Supplementary figure 6**: Risk of study bias analyzed with the SYRCLE tool. For each item, the percentage of studies scored low/unclear/high risk of bias is shown.

**Supplementary figure 7:** Funnel plots showing publication bias for the main outcomes: inflammation markers (A), eNOS (B), oxidative stress (C) in WT mice and leukocytes infiltration in ApoE^-/-^ mice (D). The reported p-values correspond to the Egger regression test.

**Supplementary figure 8**: Tercile distribution of studies and calculation of hypoxic score. The studies were distributed depending on the settings for each of the five hypoxic cycles parameters: (A) FiO_2_ during hypoxic phase (in %), (B) duration of each hypoxic phase (in seconds), (C) duration of IH exposure per day (in hours), (D) total duration of IH exposure (in days), and (E) duration of reoxygenation (seconds). The values were divided into 2 or 3 parts based on the calculation of halves or terciles and a score between 1 and 3 was assigned according to the distribution of studies into these 2 or 3 parts for the five parameters studied, in order to calculate a hypoxic score (F).

**Supplementary figure 9**: Forest plot showing the SMDs for the main outcomes after sensitivity analysis (exclusion of the studies with imputed SD).
