# Supplementary material for: Oxidative and Inflammatory Mechanisms Induced by Intermittent Hypoxia Leading to Vascular Alterations in Rodents: A Systematic Review and Meta‐Analysis
Source: Oxid Med Cell Longev. 2026 Jan 14;2026:9967028. doi: 10.1155/omcl/9967028 (PMC12802560; doi:10.1155/omcl/9967028)

Supplementary Figure 1

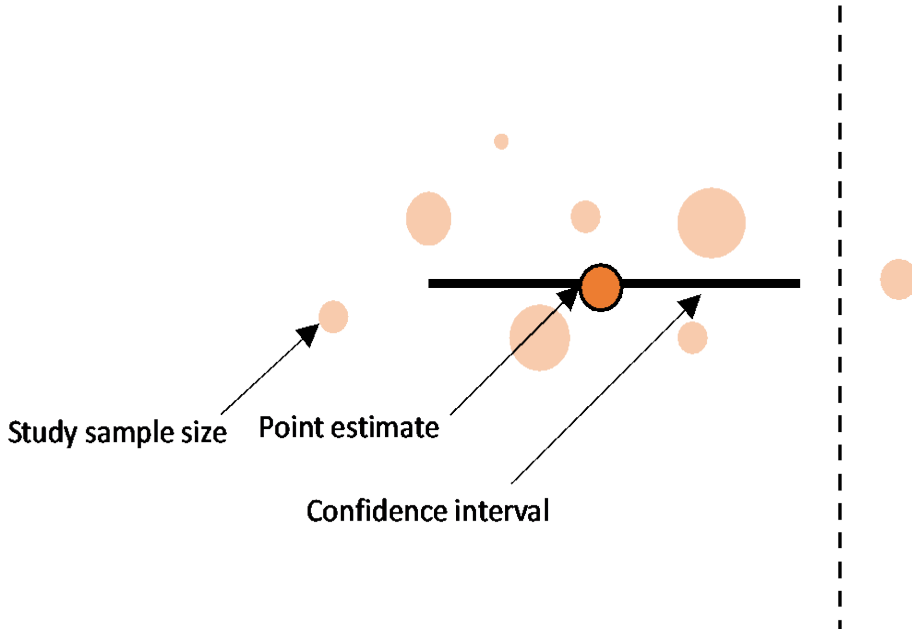

Supplementary Figure 2

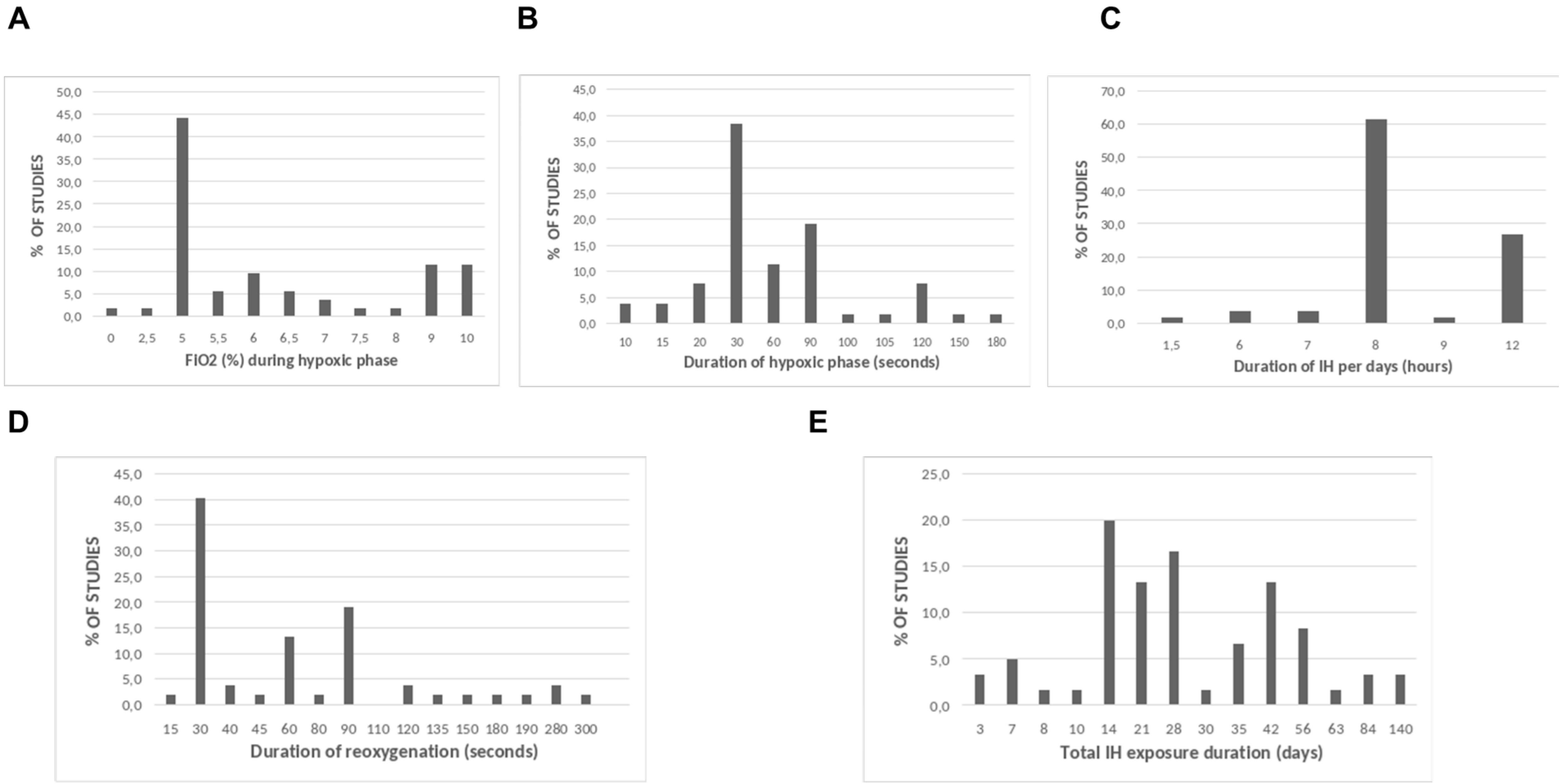

Supplementary Figure 3.

A

Study	Cluster	Experimental Total	Experimental Mean	Experimental SD	Control Total	Control Mean	Control SD	SMD	95%-CI	Weight
Arnaud C and Bouyon S_2018_1	32	7	1.34	0.6614	7	0.99	0.1587	0.68	[-0.41; 1.77]	2.6%
Arnaud C and Bouyon S_2018_2	32	4	184.00	30.0000	4	100.00	12.0000	3.19	[0.65; 5.74]	0.5%
Arnaud C and Bouyon S_2018_3	32	10	171.00	60.0833	10	100.00	63.2456	1.10	[0.15; 2.06]	3.4%
Arnaud C_2011_1	23	4	2.30	0.6000	4	1.30	0.2000	1.94	[0.04; 3.84]	3.5%
Arnaud C_2011_2	23	4	1.32	0.1800	4	0.86	0.0900	2.81	[0.47; 5.14]	2.3%
Bi R and Dai Y_2019_1	38	10	3.40	0.2800	10	1.00	0.2500	8.66	[5.56; 11.76]	0.9%
Bi R and Dai Y_2019_2	38	10	2.60	0.3000	10	1.00	0.2000	6.01	[3.77; 8.25]	1.8%
Bi R and Dai Y_2019_4	38	10	1.79	0.2000	10	1.00	0.1900	3.88	[2.28; 5.47]	3.5%
Castro-Grattoni AL_2021_10	31	8	3.40	8.4853	8	1.00	8.4853	0.27	[-0.72; 1.25]	0.7%
Castro-Grattoni AL_2021_11	31	8	1.00	1.4142	8	1.00	1.4142	0.00	[-0.98; 0.98]	0.7%
Castro-Grattoni AL_2021_12	31	8	1.00	1.4142	8	1.00	1.4142	0.00	[-0.98; 0.98]	0.7%
Castro-Grattoni AL_2021_13	31	8	0.75	1.8385	8	1.00	1.8385	-0.13	[-1.11; 0.85]	0.7%
Castro-Grattoni AL_2021_14	31	8	0.75	1.8385	8	1.00	1.8385	-0.13	[-1.11; 0.85]	0.7%
Castro-Grattoni AL_2021_5	31	8	0.70	1.1879	8	1.00	1.1879	-0.24	[-1.22; 0.75]	0.7%
Castro-Grattoni AL_2021_6	31	8	0.75	1.3294	8	1.00	1.3294	-0.18	[-1.16; 0.80]	0.7%
Castro-Grattoni AL_2021_7	31	8	0.49	1.1314	8	1.00	1.1314	-0.43	[-1.42; 0.57]	0.7%
Castro-Grattoni AL_2021_8	31	8	0.49	1.1314	8	1.00	1.1314	-0.43	[-1.42; 0.57]	0.7%
Castro-Grattoni AL_2021_9	31	8	3.40	8.4853	8	1.00	8.4853	0.27	[-0.72; 1.25]	0.7%
Del Rio R_2010_1	25	8	151.30	5.6569	6	100.00	5.6569	8.49	[4.67; 12.31]	3.4%
Gras E_2016_1	9	15	125.20	21.6887	15	100.00	16.6538	1.27	[0.47; 2.06]	6.4%
Hernandez-Bustabad A_2022_1	34	4	0.55	0.1400	4	0.30	0.2200	1.18	[-0.42; 2.77]	2.8%
Hernandez-Bustabad A_2022_2	34	4	2.43	0.2400	4	2.29	0.2200	0.53	[-0.90; 1.96]	3.5%
Hung MW-B_2013_1	18	6	0.29	0.1568	6	0.07	0.0686	1.75	[0.34; 3.16]	2.7%
Hung MW-C_2013_2	18	6	0.25	0.1029	6	0.05	0.0808	1.99	[0.51; 3.48]	2.4%
Hung MW-D_2013_3	18	6	0.47	0.1029	6	0.16	0.0465	3.57	[1.51; 5.63]	1.3%
J. Zhu et al_2019_1	1	15	0.62	0.0600	15	0.30	0.0300	6.56	[4.65; 8.48]	5.4%
Li D_2023_4	45	3	76.67	3.1004	3	67.69	1.7667	2.84	[-0.19; 5.87]	1.8%
Li D_2023_5	45	3	81.96	4.4514	3	65.27	4.8844	2.85	[-0.19; 5.89]	1.8%
Li D_2023_6	45	3	74.67	2.7886	3	66.03	2.4422	2.63	[-0.24; 5.50]	2.0%
Philippi NR-A_2010_1	26	6	65.90	47.4000	6	58.00	39.6000	0.17	[-0.97; 1.30]	0.8%
Philippi NR-B_2010_2	26	6	71.90	29.4000	6	67.90	30.0000	0.12	[-1.01; 1.26]	0.8%
Philippi NR-C_2010_3	26	6	66.30	37.8000	6	69.90	34.8000	-0.09	[-1.22; 1.04]	0.8%
Philippi NR-D_2010_4	26	6	77.70	43.2000	6	88.00	59.4000	-0.18	[-1.32; 0.95]	0.8%
Philippi NR-E_2010_5	26	6	57.50	9.5530	6	59.20	9.0631	-0.17	[-1.30; 0.97]	0.8%
Philippi NR-F_2010_6	26	6	65.40	9.0631	6	69.80	12.0025	-0.38	[-1.53; 0.76]	0.8%
Philippi NR-G_2010_7	26	6	70.50	13.2272	6	74.60	10.7778	-0.31	[-1.46; 0.83]	0.8%
Philippi NR-H_2010_8	26	6	63.40	9.3081	6	67.20	3.9192	-0.49	[-1.65; 0.67]	0.8%
Poulain L_2015_1	10	8	260.00	169.7056	8	100.00	84.8528	1.13	[0.05; 2.20]	6.2%
Song D-A_2012_1	20	5	1.02	0.1789	5	0.16	0.0894	5.49	[2.21; 8.77]	3.9%
Wang J and Zhang H_2023_1	41	7	2.76	1.0900	6	1.00	0.4700	1.89	[0.50; 3.28]	3.3%
Wang J and Zhang H_2023_2	41	7	1.71	0.4000	6	1.00	0.1200	2.16	[0.69; 3.62]	3.0%
Zhang S_2024_2	44	6	2.36	0.2300	6	1.56	0.1600	3.73	[1.60; 5.85]	5.2%
Zhang Y-A_2017_1	5	8	0.11	0.0424	8	0.06	0.0212	1.41	[0.28; 2.54]	2.1%
Zhang Y-B_2017_2	5	8	0.18	0.0509	8	0.12	0.0255	1.41	[0.28; 2.54]	2.1%
Zhang Y-C_2017_3	5	8	0.19	0.0424	8	0.06	0.0212	3.66	[1.91; 5.41]	0.9%
Zhang Y-D_2017_4	5	8	0.24	0.0509	8	0.15	0.0255	2.11	[0.83; 3.40]	1.6%
Zhou S-A_2014_1	13	6	1.05	0.1500	6	1.00	0.2100	0.25	[-0.89; 1.39]	2.3%
Zhou S-B_2014_2	13	6	0.94	0.0500	6	1.00	0.0200	-1.45	[-2.78; -0.12]	1.7%
Zhou S-C_2014_3	13	6	1.05	0.1000	6	1.00	0.2100	0.28	[-0.86; 1.42]	2.3%
Zhou S-D_2014_4	13	6	1.68	0.1000	6	1.00	0.1000	6.27	[3.05; 9.50]	0.3%
**Random effects model**		**349**			**345**			**2.32**	**[1.30; 3.33]**	**100.0%**

Heterogeneity: $I^2 = 80.7\%$, $\tau^2 = 3.9835$, $p < 0.0001$

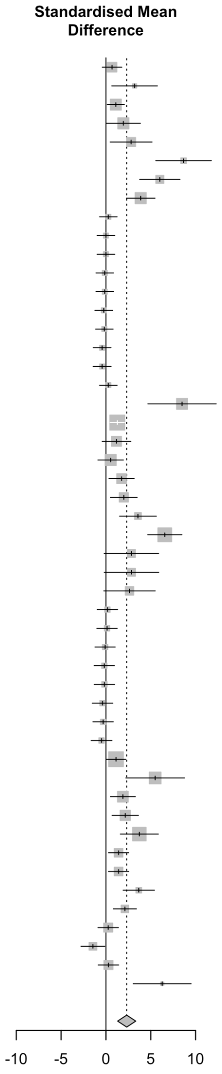

B

Study	Cluster	Experimental Total	Experimental Mean	Experimental SD	Control Total	Control Mean	Control SD	SMD	95%-CI	Weight
Arnaud C_2011_1	23	9	2.70	1.5000	9	1.40	1.5000	0.83	[-0.15; 1.80]	22.0%
Cortese R_2017_1	6	6	5.10	0.2000	6	4.10	0.1000	5.84	[2.80; 8.87]	14.7%
Gileles-Hillel A_2014_1	12	12	8.10	1.0392	12	6.40	1.0392	1.58	[0.64; 2.52]	22.1%
Song D-A_2012_1	20	5	12.20	2.4597	5	1.50	2.4597	3.93	[1.43; 6.43]	12.3%
Song D-B_2012_2	20	5	29.50	2.4597	5	14.60	2.0125	5.99	[2.45; 9.52]	6.1%
Xu C_2023_10	46	4	227.00	90.0000	4	191.00	32.0000	0.46	[-0.96; 1.88]	3.0%
Xu C_2023_11	46	4	2.00	0.2000	4	1.00	2.2000	0.56	[-0.88; 1.99]	2.9%
Xu C_2023_12	46	4	2.10	0.4000	4	1.00	2.2000	0.60	[-0.84; 2.05]	2.9%
Xu C_2023_13	46	4	1.30	0.4600	4	1.00	2.2000	0.16	[-1.23; 1.55]	3.1%
Xu C_2023_14	46	4	2.30	1.0000	4	1.00	2.2000	0.66	[-0.79; 2.12]	2.9%
Xu C_2023_7	46	4	671.00	324.0000	4	247.00	82.0000	1.56	[-0.18; 3.29]	2.0%
Xu C_2023_8	46	4	100.00	20.0000	4	65.00	82.0000	0.51	[-0.92; 1.94]	3.0%
Xu C_2023_9	46	4	45.00	62.0000	4	50.50	40.0000	-0.09	[-1.48; 1.30]	3.1%
**Random effects model**		**69**			**69**			**2.35**	**[0.44; 4.26]**	**100.0%**

Heterogeneity: $I^2 = 60.4\%$, $\tau^2 = 4.0784$, $p = 0.0025$

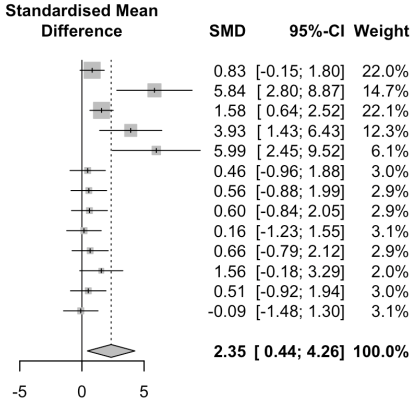

Supplementary Figure 4

A

Study	Cluster	Experimental Total	Experimental Mean	Experimental SD	Control Total	Control Mean	Control SD	SMD	95%-CI	Weight
Arnaud C and Bouyon S_2018_1	32	7	-9.30	4.7624	7	-2.60	2.6458	-1.63	[-2.89; -0.37]	1.9%
Arnaud C and Bouyon S_2018_2	32	7	-6.40	3.4395	7	-2.30	1.8520	-1.39	[-2.60; -0.18]	2.0%
Arnaud C and Bouyon S_2018_3	32	7	-1929.00	783.1424	7	-987.00	380.9882	-1.43	[-2.65; -0.21]	2.0%
Badran M and Abuyassin B_2019_1	36	10	258053.00	38225.0000	10	100573.00	22494.0000	4.81	[2.94; 6.68]	3.0%
Badran M and Abuyassin B_2019_2	36	5	1.20	0.1000	4	0.91	0.0600	3.01	[0.77; 5.26]	2.1%
Castro-Grattoni AL_2021_1	31	8	-0.32	0.7071	8	-1.00	0.7071	0.91	[-0.13; 1.95]	1.4%
Castro-Grattoni AL_2021_2	31	8	-0.32	0.7071	8	-1.00	0.7071	0.91	[-0.13; 1.95]	1.4%
Castro-Grattoni AL_2021_3	31	8	-0.60	0.9899	8	-1.00	0.9899	0.38	[-0.61; 1.37]	1.6%
Castro-Grattoni AL_2021_4	31	8	-0.60	0.9899	8	-1.00	0.9899	0.38	[-0.61; 1.37]	1.6%
Hung MW-B_2013_1	18	6	0.19	0.0147	6	0.12	0.2205	0.41	[-0.74; 1.56]	2.1%
Hung MW-C_2013_2	18	6	0.40	0.1225	6	0.17	0.0980	1.91	[0.45; 3.37]	1.4%
Hung MW-D_2013_3	18	6	0.38	0.2449	6	0.12	0.1715	1.13	[-0.12; 2.39]	1.8%
Hung MW-E_2013_4	18	6	-0.20	0.0490	6	-0.50	0.0735	4.43	[2.02; 6.85]	0.5%
Lan XF_2017_1	4	12	-17.50	5.1962	12	-25.50	8.6603	1.08	[0.21; 1.95]	5.6%
Li D_2023_1	45	3	64.44	0.8833	3	60.68	1.1778	2.88	[-0.19; 5.95]	1.9%
Li D_2023_2	45	3	-73.13	4.3994	3	-95.97	4.9190	3.91	[0.02; 7.80]	1.2%
Li D_2023_3	45	3	82.72	2.5634	3	99.06	4.8671	-3.35	[-6.79; 0.09]	1.5%
Marcus NJ-A_2012_1	21	6	3.90	0.9798	6	3.90	1.2247	0.00	[-1.13; 1.13]	5.4%
Mentek M and Morand J_2018_1	30	8	85.00	20.0000	7	66.00	12.0000	1.06	[-0.04; 2.17]	5.4%
Ozcan B_2022_1	43	5	1.00	0.1700	5	1.00	0.2700	0.00	[-1.24; 1.24]	1.3%
Ozcan B_2022_2	43	5	680.00	437.0000	5	649.00	521.0000	0.06	[-1.18; 1.30]	1.3%
Ozcan B_2022_3	43	5	258.00	231.0000	5	524.00	287.0000	-0.92	[-2.26; 0.42]	1.1%
Ozcan B_2022_4	43	5	-443.00	254.0000	5	-316.00	212.0000	-0.49	[-1.76; 0.78]	1.2%
Ozcan B_2022_5	43	4	5.00	1.6000	4	3.60	1.6000	0.76	[-0.72; 2.24]	0.9%
Peng YJ_2012_1	22	6	2.60	0.2449	6	1.10	0.2449	5.65	[2.70; 8.60]	3.5%
Philippi NR-E_2010_5	26	6	42.00	9.3081	6	52.00	14.2070	-0.77	[-1.96; 0.42]	0.7%
Philippi NR-F_2010_6	26	6	78.90	15.6767	6	83.70	15.6767	-0.28	[-1.42; 0.86]	0.8%
Philippi NR-G_2010_7	26	6	48.40	14.2070	6	54.30	19.5959	-0.32	[-1.46; 0.82]	0.8%
Philippi NR-H_2010_8	26	6	50.50	19.1060	6	43.20	17.6363	0.37	[-0.78; 1.51]	0.8%
Philippi NR-I_2010_9	26	6	45.90	8.5732	6	52.00	14.9419	-0.46	[-1.62; 0.69]	0.8%
Philippi NR-J_2010_10	26	6	63.80	14.2070	6	75.60	12.9823	-0.80	[-2.00; 0.40]	0.7%
Philippi NR-K_2010_11	26	6	42.70	12.0025	6	44.80	13.9621	-0.15	[-1.28; 0.99]	0.8%
Philippi NR-L_2010_12	26	6	45.50	12.7373	6	42.60	16.6565	0.18	[-0.95; 1.32]	0.8%
Ribon-Demars A and Pialoux V_2019_1	35	10	1.97	1.2300	14	1.97	1.0500	0.00	[-0.81; 0.81]	0.6%
Ribon-Demars A and Pialoux V_2019_10	35	7	-14.47	5.4500	8	-12.63	12.0800	-0.18	[-1.20; 0.84]	0.4%
Ribon-Demars A and Pialoux V_2019_11	35	7	9.41	3.0900	8	9.41	6.5800	0.00	[-1.01; 1.01]	0.4%
Ribon-Demars A and Pialoux V_2019_12	35	7	120.26	125.8200	8	117.64	20.9600	0.03	[-0.99; 1.04]	0.4%
Ribon-Demars A and Pialoux V_2019_2	35	10	1.92	1.2000	14	2.61	0.2500	-0.84	[-1.69; 0.01]	0.6%
Ribon-Demars A and Pialoux V_2019_3	35	10	-4.75	1.9300	14	-5.65	2.8100	0.35	[-0.47; 1.17]	0.6%
Ribon-Demars A and Pialoux V_2019_4	35	10	-3.23	5.5900	14	-3.02	4.1400	-0.04	[-0.85; 0.77]	0.6%
Ribon-Demars A and Pialoux V_2019_5	35	10	13.31	12.7800	14	6.83	0.8000	0.76	[-0.08; 1.61]	0.6%
Ribon-Demars A and Pialoux V_2019_6	35	10	80.00	51.1000	14	151.00	37.7000	-1.57	[-2.51; -0.62]	0.5%
Ribon-Demars A and Pialoux V_2019_7	35	7	2.33	2.0200	8	2.29	0.7700	0.03	[-0.99; 1.04]	0.4%
Ribon-Demars A and Pialoux V_2019_8	35	7	2.20	1.4100	8	2.26	0.8000	-0.05	[-1.06; 0.96]	0.4%
Ribon-Demars A and Pialoux V_2019_9	35	7	-3.19	3.0800	8	-6.29	2.0800	1.13	[0.01; 2.24]	0.4%
Rubies C and Dantas AP_2019_8	37	15	0.31	0.0966	12	0.24	0.0601	0.88	[0.08; 1.68]	4.1%
Rubies C and Dantas AP_2019_9	37	5	2.39	1.2061	7	0.98	0.4040	1.58	[0.21; 2.96]	1.7%
Schulz R-B_2014_1	16	5	8.00	3.1305	5	5.60	4.0249	0.60	[-0.68; 1.88]	2.0%
Schulz R-C_2014_2	16	5	13.60	1.1180	5	11.50	4.0249	0.64	[-0.65; 1.93]	2.0%
Schulz R-D_2014_3	16	5	14.50	1.7889	5	12.80	1.5652	0.91	[-0.43; 2.25]	1.8%
Yan YR and Zhang L_2021_1	29	7	33.00	7.9373	7	9.00	2.6458	3.80	[1.85; 5.74]	2.2%
Yan YR and Zhang L_2021_2	29	7	-144.00	18.5203	7	-228.00	34.3948	2.85	[1.23; 4.46]	3.1%
Zhang S_2024_1	44	6	-21.00	2.3000	6	-46.10	4.3000	6.72	[3.29; 10.15]	2.2%
Zhang S_2024_2	44	6	5.72	0.6200	6	1.79	0.2000	7.87	[3.91; 11.83]	1.7%
Zhang Y-A_2017_1	5	8	0.24	0.0566	8	0.11	0.0283	2.75	[1.28; 4.21]	1.3%
Zhang Y-B_2017_2	5	8	57.10	40.4465	8	0.00	20.0818	1.69	[0.50; 2.88]	1.9%
Zhang Y-C_2017_3	5	8	0.41	0.1131	8	0.13	0.0283	3.21	[1.61; 4.81]	1.1%
Zhang Y-D_2017_4	5	8	128.60	60.5283	8	14.30	40.4465	2.10	[0.81; 3.38]	1.6%
Zhao F and Meng Y_2022_2	40	3	3.73	0.4700	3	1.06	0.4100	4.83	[0.16; 9.50]	2.1%
Zhou S-A_2014_1	13	6	1.05	0.2600	6	1.00	0.0500	0.25	[-0.89; 1.38]	1.9%
Zhou S-B_2014_2	13	6	1.05	0.0500	6	1.00	0.1500	0.41	[-0.74; 1.56]	1.9%
Zhou S-C_2014_3	13	6	1.05	0.1000	6	1.00	0.1000	0.46	[-0.69; 1.61]	1.9%
Zhou S-D_2014_4	13	6	1.89	0.1000	6	1.00	0.1000	8.21	[4.10; 12.33]	0.2%
**Random effects model**		**427**			**454**			**1.41**	**[0.56; 2.25]**	**100.0%**

Heterogeneity: $I^2 = 75.6\%$, $\tau^2 = 3.1066$, $p < 0.0001$

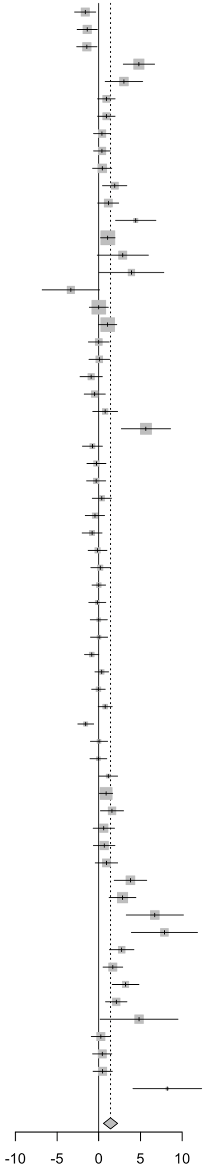

B

Study	Cluster	Experimental Total	Experimental Mean	Experimental SD	Control Total	Control Mean	Control SD	SMD	95%-CI	Weight
Arnaud C and Bouyon S_2018_1	32	7	0.95	0.2381	7	1.00	0.2117	-0.21	[-1.26; 0.84]	4.6%
Badran M-A_2014_1	14	6	1.26	0.3184	6	1.10	0.2449	0.52	[-0.64; 1.68]	3.5%
Badran M-B_2014_2	14	6	1.25	0.4654	6	1.18	0.5144	0.13	[-1.00; 1.27]	3.5%
Castro-Grattoni AL_2021_5	31	8	0.50	1.1314	8	1.00	1.1314	-0.42	[-1.41; 0.58]	3.5%
Castro-Grattoni AL_2021_6	31	8	0.50	1.1314	8	1.00	1.1314	-0.42	[-1.41; 0.58]	3.5%
Chen L-C_2017_1	3	8	0.36	0.0453	8	0.52	0.0453	-3.34	[-4.99; -1.70]	4.3%
Guo QH-E_2013_1	19	6	69.40	9.3081	6	104.20	10.7778	-3.19	[-5.10; -1.28]	4.1%
Hernandez-Bustabad A_2022_1	34	4	1.23	0.0700	4	0.97	0.0600	3.40	[0.74; 6.06]	2.3%
Hernandez-Bustabad A_2022_2	34	4	0.48	0.2600	4	0.64	0.0600	-0.74	[-2.21; 0.73]	2.9%
Hernandez-Bustabad A_2022_3	34	4	0.55	0.1000	4	0.64	0.1200	-0.71	[-2.17; 0.76]	2.9%
Hung MW-B_2013_1	18	6	0.70	0.1225	6	1.95	0.1225	-9.42	[-14.09; -4.74]	2.4%
J. Zhu et al_2019_1	1	15	0.30	0.0500	15	0.55	0.0100	-6.75	[-8.71; -4.79]	4.1%
Kaczmarek E_2013_1	17	7	0.48	0.1100	7	0.30	0.0670	1.78	[0.48; 3.08]	4.5%
Krause BJ_2015_1	11	6	0.50	0.2449	6	2.50	1.2247	-2.09	[-3.60; -0.57]	4.3%
Li JR-A_2018_1	2	6	0.60	0.1960	6	1.10	0.2449	-2.08	[-3.59; -0.57]	4.3%
Philippi NR-A_2010_1	26	6	67.20	6.3687	6	72.10	16.6565	-0.36	[-1.50; 0.79]	2.4%
Philippi NR-B_2010_2	26	6	72.20	10.0429	6	86.10	10.0429	-1.28	[-2.57; 0.01]	2.4%
Philippi NR-C_2010_3	26	6	76.00	13.4722	6	70.30	7.1035	0.49	[-0.67; 1.64]	2.4%
Philippi NR-D_2010_4	26	6	70.30	15.1868	6	61.20	6.3687	0.72	[-0.46; 1.90]	2.4%
Rubies C and Dantas AP_2019_8	37	5	0.59	0.2878	7	0.99	0.3937	-1.06	[-2.31; 0.20]	3.4%
Rubies C and Dantas AP_2019_9	37	6	66.15	11.4563	7	99.22	68.9324	-0.60	[-1.72; 0.53]	3.5%
Snow JB-A_2008_1	27	4	228.60	64.2000	4	64.30	50.0000	2.48	[0.32; 4.64]	4.0%
Tahawi Z-A_2001_1	28	8	0.85	0.0849	8	0.77	0.1414	0.65	[-0.36; 1.66]	4.6%
Wang JW_2017_1	8	6	0.70	0.0980	6	1.25	0.1225	-4.58	[-7.05; -2.10]	3.8%
Yang R_2011_1	24	6	143.00	44.0908	6	100.00	41.6413	0.93	[-0.29; 2.14]	4.5%
Zhao F and Lu M and Wang H_2023_2	42	3	6.38	1.3500	3	20.38	2.5600	-5.46	[-10.67; -0.25]	2.0%
Zhao F and Lu M and Wang H_2023_3	42	3	0.55	0.0800	3	1.41	0.0700	-9.13	[-17.57; -0.69]	1.0%
Zhou S-A_2014_1	13	6	1.89	0.1500	6	1.00	0.1000	6.44	[3.14; 9.75]	1.7%
Zhou S-B_2014_2	13	6	1.05	0.1800	6	1.00	0.1500	0.28	[-0.86; 1.42]	2.6%
Zhou S-C_2014_3	13	6	0.94	0.1000	6	1.00	0.1000	-0.55	[-1.72; 0.61]	2.6%
Zhou S-D_2014_4	13	6	0.52	0.1000	6	1.00	0.1000	-4.43	[-6.84; -2.01]	2.1%
**Random effects model**		**190**			**193**			**-1.12**	**[-2.16; -0.09]**	**100.0%**

Heterogeneity: $I^2 = 83.2\%$, $\tau^2 = 5.8154$, $p < 0.0001$

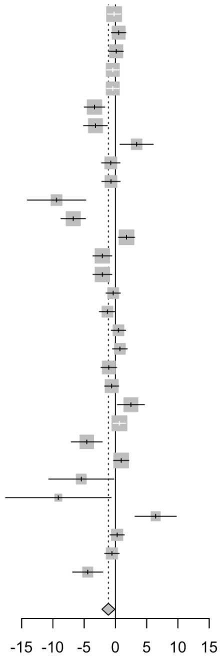

C

Study	Cluster	Experimental Total	Experimental Mean	Experimental SD	Control Total	Control Mean	Control SD	SMD	95%-CI	Weight
Bi R and Dai Y_2019_1	38	10	2.50	0.3000	10	0.80	0.2000	6.39	[4.02; 8.75]	14.2%
Bi R and Dai Y_2019_2	38	10	3.20	0.4000	10	0.99	0.2000	6.69	[4.23; 9.15]	13.1%
Ding H and Huang J_2020_1	39	6	10.79	1.5400	6	5.54	0.8900	3.85	[1.68; 6.03]	23.2%
Yan YR and Zhang L_2021_1	29	7	1.90	0.2646	7	1.00	0.2646	3.18	[1.45; 4.91]	27.0%
Zhao Y_2025_2	47	3	-0.80	0.0400	3	-1.00	0.0400	3.99	[0.03; 7.95]	7.4%
Zhao Y_2025_3	47	3	1.31	0.1000	3	1.00	0.1000	2.47	[-0.28; 5.23]	15.2%
**Random effects model**		**39**			**39**			**4.20**	**[2.50; 5.90]**	**100.0%**

Heterogeneity: $I^2 = 50.4\%$, $\tau^2 = 2.0032$, $p = 0.0728$

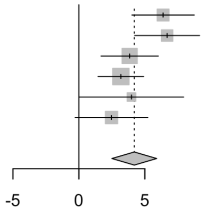

Supplementary Figure 5

A

Study	Cluster	Experimental Total	Experimental Mean	Experimental SD	Control Total	Control Mean	Control SD	SMD	95%-CI	Weight
Fang G-A_2012_1	47	5	10.20	2.0125	5	1.10	2.0125	4.08	[1.51; 6.66]	31.6%
Xu C and Xu J_2021_1	50	6	1.11	0.0680	6	1.00	0.0340	1.89	[0.44; 3.34]	47.2%
Zeng X and Guo R_2018_1	49	3	0.03	0.0035	3	0.01	0.0017	5.83	[0.30; 11.35]	9.2%
Zeng X and Guo R_2018_2	49	3	1.49	0.0693	3	0.93	0.1039	5.06	[0.19; 9.92]	11.9%
**Random effects model**		**17**			**17**			**3.32**	**[1.25; 5.40]**	**100.0%**

Heterogeneity: $I^2 = 32\%$, $\tau^2 = 1.8268$, $p = 0.22$

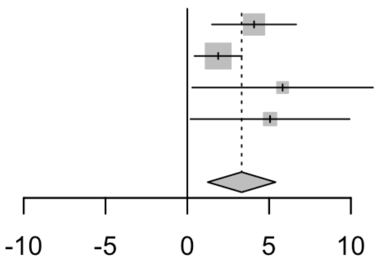

B

Study	Cluster	Experimental Total	Experimental Mean	Experimental SD	Control Total	Control Mean	Control SD	SMD	95%-CI	Weight
Arnaud C-C_2011_1	48	8	21.40	8.2024	9	22.30	15.3000	-0.07	[-1.02; 0.88]	14.0%
Arnaud C-F_2011_2	48	8	7.50	1.6971	9	4.30	1.8000	1.73	[0.57; 2.89]	12.3%
Poulain L-A_2014_1	46	5	3.81	1.2522	5	5.40	3.1305	-0.60	[-1.89; 0.68]	13.0%
Poulain L-B_2014_2	46	5	0.29	0.0894	5	0.25	0.1118	0.36	[-0.90; 1.61]	13.2%
Tan H_2023_6	51	8	25.20	2.4000	8	5.51	1.7300	8.90	[5.24; 12.55]	20.6%
Xu C and Xu J_2021_1	50	6	4.77	0.9100	6	4.67	0.9100	0.10	[-1.03; 1.23]	5.9%
Xu C and Xu J_2021_2	50	6	7.02	2.2100	6	6.46	1.6600	0.26	[-0.87; 1.40]	5.9%
Xu C and Xu J_2021_3	50	6	1.22	0.0300	6	1.01	0.0900	2.89	[1.09; 4.68]	3.8%
Xu C and Xu J_2021_4	50	6	1.18	0.1600	6	1.01	0.0800	1.24	[-0.04; 2.52]	5.4%
Xu C and Xu J_2021_5	50	6	1.08	0.2100	6	1.01	0.0900	0.40	[-0.75; 1.55]	5.8%
**Random effects model**		**64**			**66**			**2.23**	**[-1.39; 5.85]**	**100.0%**

Heterogeneity: $I^2 = 76\%$, $\tau^2 = 13.1001$, $p < 0.01$

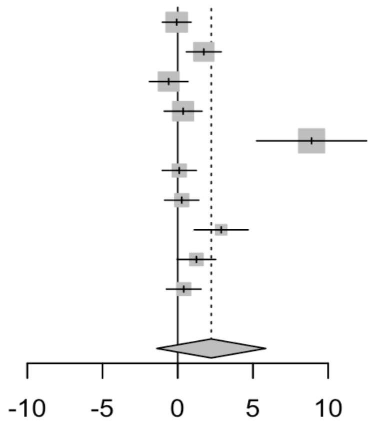

C

Study	Experimental Total	Experimental Mean	Experimental SD	Control Total	Control Mean	Control SD	SMD	95%-CI	Weight
Kato R_2015_1	8	21.00	2.0000	8	7.50	0.7000	8.52	[5.01; 12.03]	30.2%
Tuleta I_2014_1	9	1750.00	3000.0000	9	208.30	249.9000	0.69	[-0.27; 1.65]	35.0%
Xu C and Xu J_2021_1	6	2.03	0.8800	6	1.89	0.7200	0.16	[-0.97; 1.29]	34.8%
**Random effects model**	**23**			**23**			**2.87**	**[-2.16; 7.90]**	**100.0%**

Heterogeneity: $I^2 = 90\%$, $\tau^2 = 18.5963$, $p < 0.01$

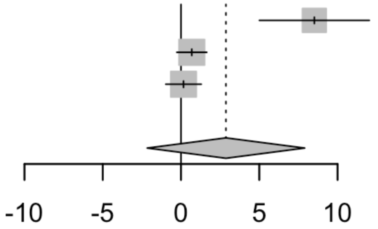

Supplementary Figure 6

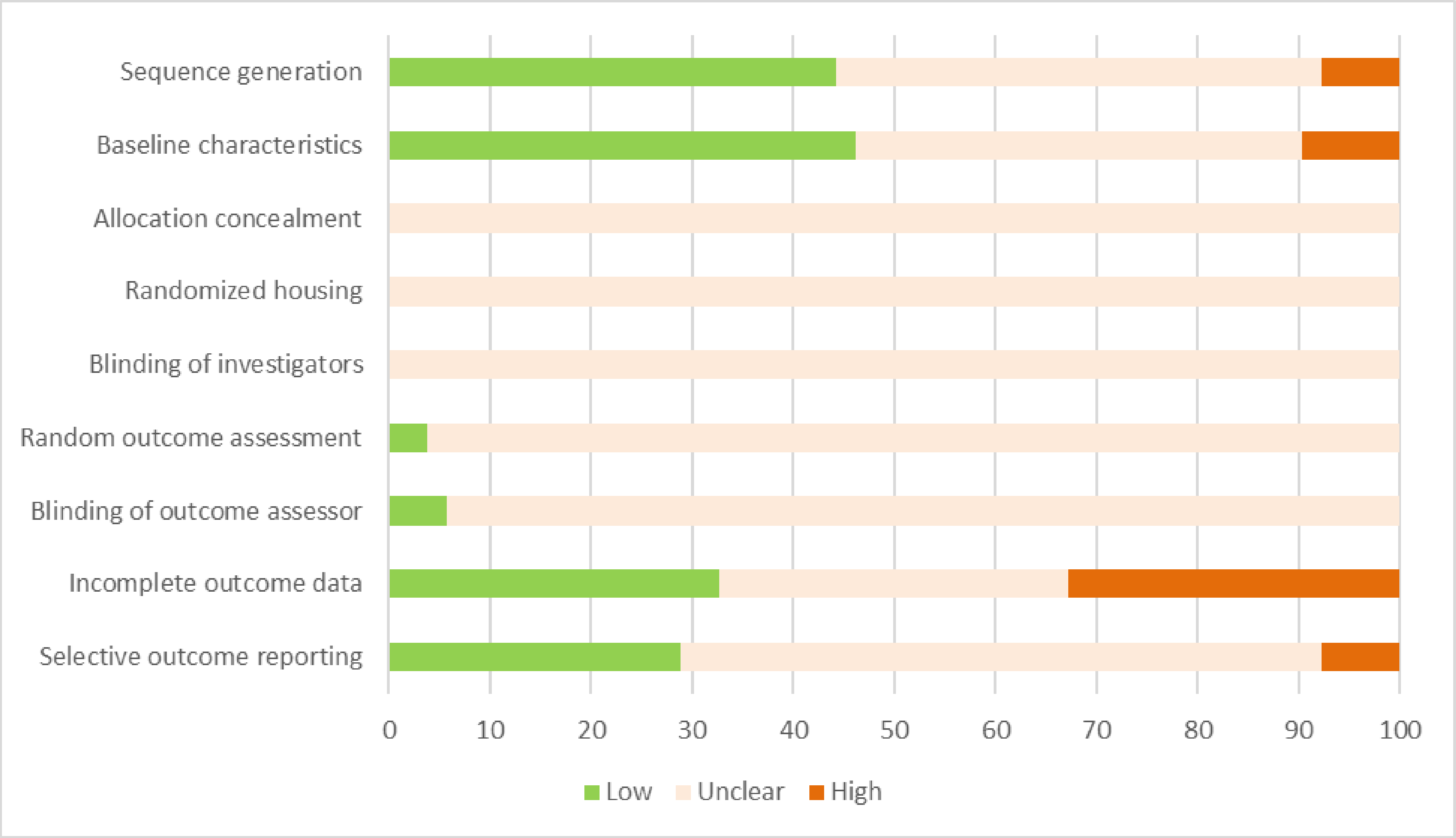

Supplementary Figure 7

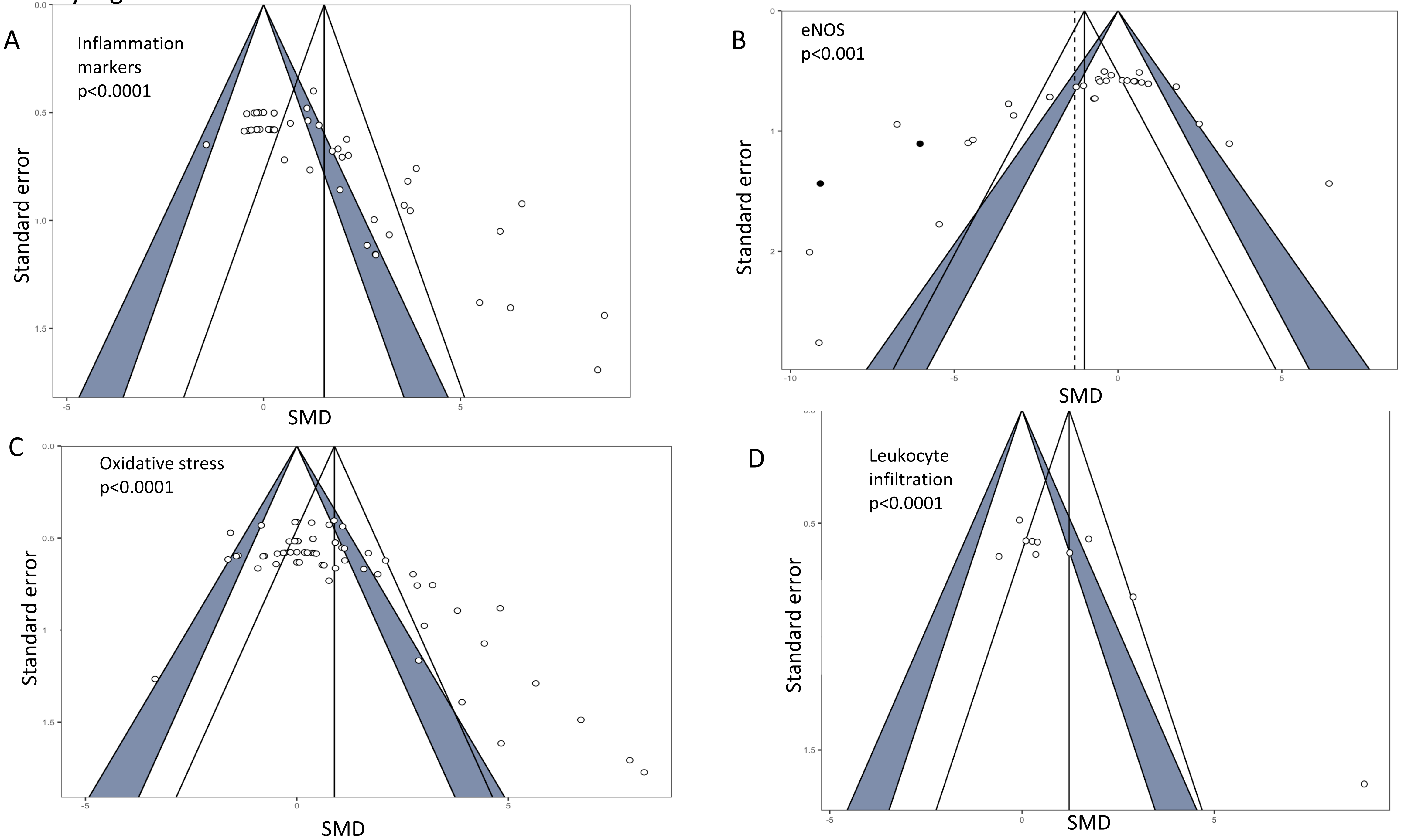

Supplementary Figure 8

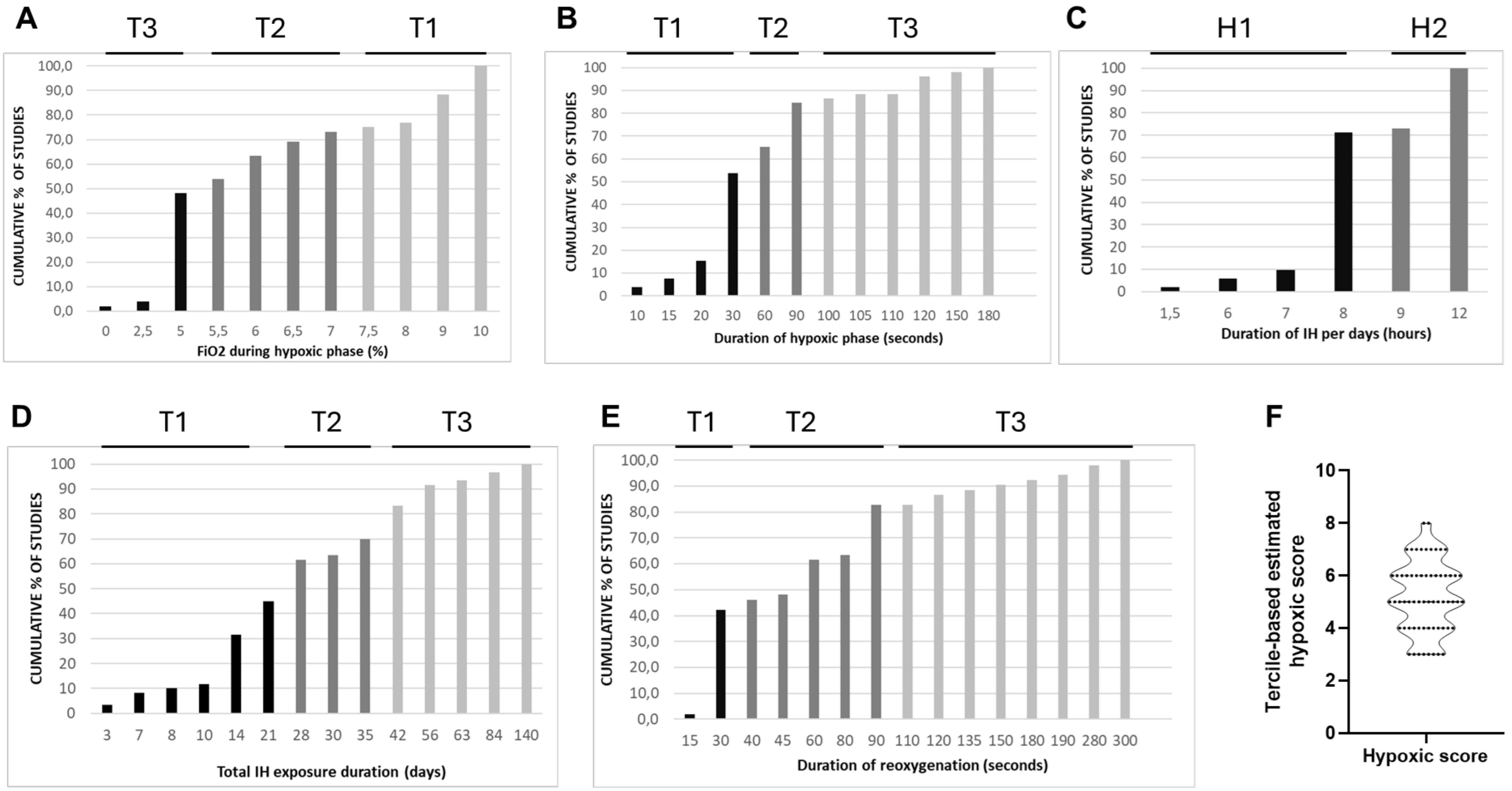

Supplementary Figure 9

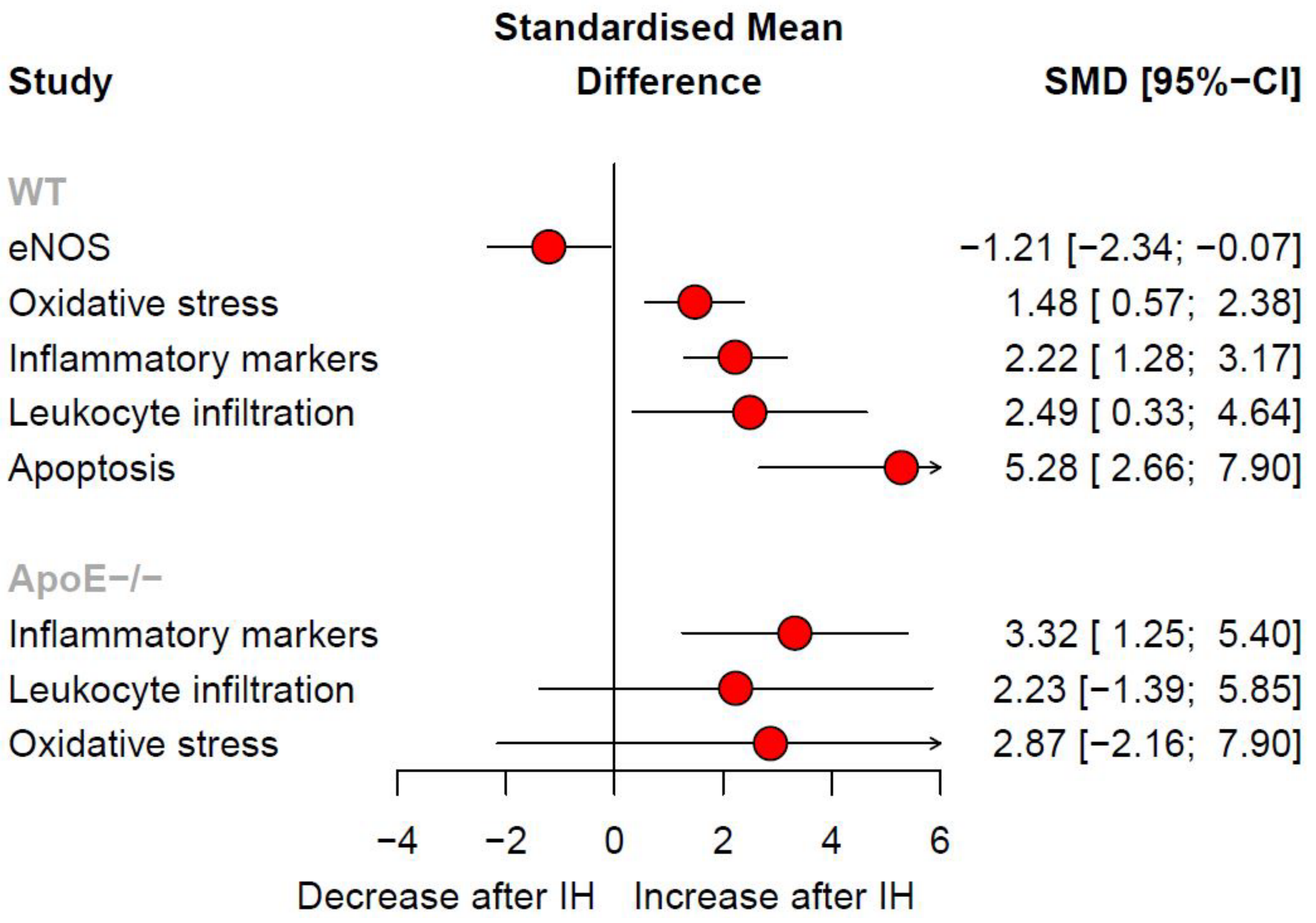

Supplement: Supplementary file 2 — Supporting Information 2 Figure S1: Orchard plot example showing the meaning of the different parts of the plot. Figure S2: Description of the number of studies included for each of the four IH parameters: (A) FiO2 during hypoxic phase (in %), (B) duration of hypoxic phase (in seconds), (C) duration of IH exposure per day (in hours), (D) duration of reoxygenation (in seconds), and (E) total duration of IH exposure (in days). Figure S3: Forest plots for (A) inflammation markers and (B) leukocyte infiltration in wild type mice. Figure S4: Forest plots for (A) oxidative stress, (B) eNOS activity, and (C) apoptosis in vascular wall in wild type mice. Figure S5: Forest plots for (A) inflammation markers, (B) leukocyte infiltration, and (C) oxidative stress in ApoE−/− mice. Figure S6: Risk of study bias analyzed with the SYRCLE tool. For each item, the percentage of studies scored low/unclear/high risk of bias is shown. Figure S7: Funnel plots showing publication bias for the main outcomes: inflammation markers (A), eNOS (B), oxidative stress (C) in WT mice, and leukocyte infiltration in ApoE−/− mice (D). The reported p‐values correspond to the Egger regression test. Figure S8: Tercile distribution of studies and calculation of hypoxic score. The studies were distributed depending on the settings for each of the five hypoxic cycles parameters: (A) FiO2 during hypoxic phase (in %), (B) duration of each hypoxic phase (in seconds), (C) duration of IH exposure per day (in hours), (D) total duration of IH exposure (in days), and (E) duration of reoxygenation (seconds). The values were divided into two or three parts based on the calculation of halves or terciles and a score between 1 and 3 was assigned according to the distribution of studies into these two or three parts for the five parameters studied, in order to calculate a hypoxic score (F). Figure S9: Forest plot showing the SMDs for the main outcomes after sensitivity analysis (exclusion of the studies with imputed SD). [file OMCL-2026-9967028-s003.pdf]
